# A Meta-analysis of Immune Parameters, Variability, and Assessment of Modal Distribution in Psychosis and Test of the Immune Subgroup Hypothesis

**DOI:** 10.1093/schbul/sby160

**Published:** 2018-11-08

**Authors:** Toby Pillinger, Emanuele F Osimo, Stefan Brugger, Valeria Mondelli, Robert A McCutcheon, Oliver D Howes

**Affiliations:** 1 Institute of Psychiatry, Psychology and Neuroscience, King’s College London, London, UK; 2 Department of Psychiatry, University of Cambridge, Cambridge, UK; 3 Cambridgeshire and Peterborough NHS Foundation Trust, Cambridge, UK; 4 Institute of Clinical Sciences, Faculty of Medicine, Imperial College London, London, UK; 5 Medical Research Council London Institute of Medical Sciences, London, UK; 6 Division of Psychiatry, University College London, London, UK; 7 National Institute for Health Research (NIHR) Mental Health Biomedical Research Centre at South London and Maudsley NHS Foundation Trust and King’s College London, London, UK

**Keywords:** inflammation, immune, psychosis, variability

## Abstract

Immune parameters are elevated in psychosis, but it is unclear whether alterations are homogenous across patients or heterogeneity exists, consistent with the hypothesis that immune alterations are specific to a subgroup of patients. To address this, we examine whether antipsychotic-naïve first-episode psychosis patients exhibit greater variability in blood cytokines, C-reactive protein, and white cell counts compared with controls, and if group mean differences persist after adjusting for skewed data and potential confounds. Databases were searched for studies reporting levels of peripheral immune parameters. Means and variances were extracted and analyzed using multivariate meta-analysis of mean and variability of differences. Outcomes were (1) variability in patients relative to controls, indexed by variability ratio (VR) and coefficient of variation ratio (CVR); (2) mean differences indexed by Hedges *g*; (3) Modal distribution of raw immune parameter data using Hartigan’s unimodality dip test. Thirty-five studies reporting on 1263 patients and 1470 controls were included. Variability of interleukin-6 (IL6) (VR = 0.19), tumor necrosis factor-α (TNFα) (VR = 0.36), interleukin-1β (VR = 0.35), interleukin-4 (VR = 0.55), and interleukin-8 (VR = 0.28) was reduced in patients. Results persisted for IL6 and IL8 after mean-scaling. Ninety-four percent and one hundred percent of raw data were unimodally distributed in psychosis and controls, respectively. Mean levels of IL6 (*g* = 0.62), TNFα (*g* = 0.56), interferon-γ (IFNγ) (*g* = 0.32), transforming growth factor-β (*g* = 0.53), and interleukin-17 (IL17) (*g* = 0.48) were elevated in psychosis. Sensitivity analyses indicated this is unlikely explained by confounders for IL6, IFNγ, and IL17. These findings show elevated cytokines in psychosis after accounting for confounds, and that the hypothesis of an immune subgroup is not supported by the variability or modal distribution.

## Introduction

Schizophrenia and related psychotic disorders have a worldwide lifetime prevalence of approximately 1%.^1^ They are leading contributors to global disease burden, partly because of inadequate response to antipsychotic treatment in many patients.^2^ A greater understanding of illness pathophysiology is required to identify novel therapeutic targets, and develop biologically informed diagnoses.^3^ Converging lines of evidence, including genetic, postmortem, and preclinical data, suggest immune dysregulation may play a role in psychosis pathogenesis.^4–22^ Supporting this, meta-analyses have shown raised levels of soluble interleukin-2 receptor (sIL2R), interleukin-6 (IL6), interleukin-8 (IL8), interleukin-10 (IL10), interferon-γ (IFNγ), transforming growth factor-β (TGFβ), tumor necrosis factor-α (TNFα), C-reactive protein (CRP) and white cell counts in both antipsychotic-naïve first episode psychosis (FEP) and chronic medicated schizophrenia compared with controls, with large effect sizes.^16–22^

It has been hypothesized that there are biological subtypes of psychosis,^3,23–25^ with immune alterations seen only in a proportion of patients and potentially linked to poor response to treatment.^26,27^ In addition to inflating effect sizes for mean differences between patients and controls, the presence of an immune subgroup would be expected to lead to increased immune parameter variability in patients. We therefore set out to assess this by performing a meta-analysis of variability, as previously employed to examine regional brain structural variability in FEP.^28^ If immune alterations are seen only in a subgroup of patients then greater immune measure variability in patients relative to controls would be predicted, reflecting heterogeneity in immune dysregulation. Conversely, if immune alterations are a core component of the pathophysiology of psychosis, reduced immune variability in patients compared with controls would be predicted, reflecting homogeneity in immune dysregulation. This approach could however fail to identify subgroups of data contained within the overall data distribution (eg, a bimodal distribution). To address this, we also set out to examine distribution of raw immune parameter data in patients to assess for a multimodal distribution. The presence of latent immune subgroups within the healthy control group^27,29^ could also influence variability analyses, thus we also set out to assess for multimodality in controls.

Mean differences in immune parameters between patients and controls reported in previous meta-analyses^16–22^ could be influenced by physiological and environmental confounders, eg, body mass index (BMI),^30,31^ smoking,^32^ age,^33^ gender,^34^ hypothalamic–pituitary–adrenal (HPA) axis activity,^35^ and ethnicity.^36^ Moreover, mean meta-analytic differences may be influenced by data-skew. Many immune parameters are physiologically present at low concentrations, and poor assay sensitivity at these levels can result in a floor effect.^37^ This results in positive skew, potentially inflating group differences.^38,39^ Indeed, over half the data sets included in previous meta-analyses examining immune parameters in psychosis show evidence of significant skew,^26,40–60^ and the Cochrane Collaboration recommends that meta-analyses based on means are appropriate only for data that are at least approximately normally distributed.^61^ Thus, we also set out to assess if mean differences in immune parameters between patients and controls were robust to sensitivity analyses focusing on studies that matched for physiological/environmental confounds, and with skewed data removed.

To our knowledge, this article represents the first variability meta-analysis of immune parameters, the first study to examine distribution of multiple raw immune parameter data sets, and the first meta-analysis of mean differences to comprehensively examine the role of confounders and data-skew in individuals with psychosis compared with controls.

## Methods

### Selection Procedures

A systematic review was performed according to Preferred Reporting Items for Systematic Reviews and Meta-Analyses (PRISMA)^62^ and Meta-Analysis of Observational Studies in Epidemiology (MOOSE)^63^ guidelines (supplementary eAppendix 1), following an a priori protocol (supplementary eAppendix 2). Two reviewers (TP and EO) independently searched the Pubmed, EMBASE, and PsycINFO databases from inception to November (week 2) 2017 using the following keywords: (lymphocytes or T-lymphocytes or B-lymphocytes or monocytes or macrophages or inflammat* or IL-* or cytokine or CRP or C-reactive protein or hs-CRP or hsCRP or interleukin* or tumour necrosis factor or transforming growth factor or interferon) and (schizophren* or psycho*) and (first episode or early or antipsychotic* or drug* or neuroleptic*). Only English-written studies were considered. Abstracts were screened, and full texts of studies retrieved. Where texts were unavailable, authors were contacted and manuscripts requested. We also requested raw data sets from authors, and where appropriate, clarification as to whether patients were antipsychotic naïve. TP and EO selected final studies for meta-analysis.

### Selection Criteria

Inclusion criteria were (1) patients with FEP, defined either as first treatment contact/patients recruited from FEP services in line with previous studies^64^; (2) antipsychotic-naïve; (3) a healthy control group; (4) studies assessing blood cytokines/cytokine receptors, C-reactive protein (CRP) (plasma/serum samples), and white cell counts. The rationale behind focusing on antipsychotic-naïve FEP was to minimize confounding effects of medication and lifestyle habits (eg, diet/exercise) associated with chronic psychotic illness^65,66^ that may directly (eg, antipsychotics)^67^ or indirectly (eg, diet/exercise)^30,31^ alter immune parameters. Exclusion criteria were (1) genetic studies (incomplete translation and post-translational modification means mRNA levels cannot be assumed to reflect protein levels)^68^; (2) in vitro studies; (3) studies examining stimulated cytokine levels; (4) substance/medication-induced psychosis; (5) absence of data allowing mean and/or standard deviation calculation.

### Recorded Variables

Data were extracted as follows: author, publication year, matching criteria, and mean (with standard deviation) measure of immune parameter (table 1 and supplementary eTable 1). See supplementary eAppendix 3 for further details.

**Table 1.** Summary of the Designs and Sample Characteristics of the Studies Included in the Meta-analyses

Study	Patient, *N*	Control, *N*	Diagnoses	Patient Age, Mean (SD)	Immune Parameter	Matching
Ajami et al^40^	8	26	Schizophrenia	Not specified	IL2, IL10, TNFα	Not specified
Akiyama^69^	14	27	Schizophrenia	34.4 (14.0)	sIL2R, IL6	Age, gender
Borovcanin et al^41^	84	35	First-episode psychosis	33.6 (8.8)	TGFβ	Age
De Berardis et al^70^	30	30	Schizophrenia (25) Schizophreniform disorder (2) Brief psychotic disorder (1) Delusional disorder (2)	25.9 (6.0)	CRP	Age, gender
Devanarayanan et al^42^	22	40	Schizophrenia	29.0 (4.0)	CRP	Age, gender
Di Nicola et al^43^	5	24	First-episode psychosis	28.1 (1.1)	IL1β, IL2, IL4, IL6, IL8, IL10, TNFα, IFNγ	Age, gender, BMI, ethnicity
Ding et al^44^	69	60	Schizophrenia	27.5 (7.8)	IL6, IL17, IFNγ	Age, gender, BMI, smoking
El Kissi et al^45^	10	27	Schizophrenia	Not specified	IL17, IFNγ, TGFβ	Age, gender
Fawzi and Said^46^	108	200	Schizophrenia	27.2 (10.6)	CRP	Age, gender, BMI, smoking, ethnicity
Fernandez-Egea et al^47^	50	50	Schizophrenia (35) Schizophreniform disorder (8) Brief psychotic disorder (4) Delusional disorder (2) Psychosis NOS (1)	29.4	IL6, CRP	Age, gender, BMI, smoking, ethnicity, cortisol
Ganguli and Rabin^60^	4	57	Schizophrenia	Not specified	sIL2R	Not specified
Ganguli et al^71^	24	110	Schizophrenia Schizoaffective disorder	Not specified	IL6	Age, gender
Ganguli et al^72^	33	33	Schizophrenia	Not specified	IL2	Age, gender
Garcia-Rizo et al^48^	75	80	First-episode (nonaffective) psychosis	28.0 (6.2)	Total lymphocyte count	Age, gender, BMI, smoking
Gattaz et al^73^	10	11	Schizophrenia	Not specified	IL2, IFNγ	Age, gender
Haring et al^49^ Balotsev et al^74^	38	37	First-episode psychosis	25.4 (0.9)	IL1β, IL2, IL4, IL6, IL8, IL10, IFNγ, TNFα	Age, gender, BMI, smoking
Hepgul et al^50^	4	45	First-episode psychosis	Not specified	CRP	Not specified
Kalmady et al^75^	25	33	Schizophrenia	29.9 (5.7)	IL6	Age, gender
Karanikas et al^51^	25	23	Schizophrenia (1) Schizophreniform disorder (16) Brief psychotic disorder (4) Psychotic disorder NOS (4)	25.5 (5.4)	IL1b, IL2, IL4, IL8, IL10, IFNγ, TNFα	Age, BMI, cortisol
Kubistova et al^52^	25	25	Schizophrenia	32.3 (7.0)	IL6, IL8, IL10, TNFα	Age, gender
Masserini et al^53^	7	37	Schizophrenia Schizophreniform disorder	Not specified	Total lymphocyte count	Not specified
Mondelli et al^26^	3	36	First-episode psychosis	Not specified	IL1β, IL2, IL4, IL6, IL8, IL10, TNFα, IFNγ	Age, gender
Noto et al^54^ Noto et al^55^ Brinholi et al^56^	156	58	Schizophrenia Schizophreniform disorder Brief psychotic disorder Psychosis NOS Mania with psychosis	26.2 (7.6)	IL4, IL6, IL10, IL17, TNFα, IFNγ	Age, gender
Petrikis et al^57^	39	39	Schizophrenia Schizophreniform disorder Brief psychotic disorder	27.0 SD not specified	IL6, IL17, TGFβ	Age, gender, BMI, smoking
Rapaport and Lohr^76^	12	14	Schizophrenia	37.9 (11.6)	sIL2R	Age, gender, ethnicity
Şimşek et al^77^	30	26	Schizophrenia Schizophreniform disorder	14.7 1.9	IL2, IL4, IL6, IL10, IL17, TNFα, IFNγ	Age, gender, BMI, smoking
Sperner-Unterweger et al^58^	21	16	Schizophrenia	26.8 (5.5)	Total lymphocyte count	Not specified
Sirota et al^59^	6	22	Schizophrenia	Not specified	sIL2R	Age, gender
Song et al^78^	83	65	Schizophrenia	27.3 (6.7)	IL1β, TNFα	Age, gender, BMI, smoking
Song et al^79^ Song et al^80^	62	60	Schizophrenia	24.7 (5.5)	IL1β, IL6, TNFα	Age, gender, BMI, smoking
Theodoropoulou et al^81^	53	62	Schizophrenia	Not specified	IL1β, IL2, TNFα	Age, gender
Xiu et al^82^	128	62	Schizophrenia	25.8 (9.4)	IL10	Age, gender, BMI, smoking, ethnicity

### Statistical Analysis

As many studies reported on several parameters, multivariate meta-analysis was used, enabling simultaneous estimation of summary effect sizes across all immune parameters, reducing risk of false positives due to multiple comparisons.^83^ For all meta-analyses, an omnibus test evaluated significance of model coefficients across immune parameters. Where the omnibus test was significant, we tested the effect separately for each parameter. An unstructured covariance matrix was used owing to uncertainty regarding immune parameter correlations in psychosis. Analyses were only performed if ≥3 studies were identified. A 2-tailed *P* value <.05 was deemed significant. All analyses were conducted using the metafor package^84^ in the R statistical programming language.^85^

### Meta-analysis of Variability

To measure variability, the natural log of the ratio of estimates of the population standard deviations for each group was calculated to give the log variability ratio (VR), as previously described (supplementary eAppendix 3).^28,86^ In biological systems, variance often scales with mean.^87^ Thus, between group differences in relative variability may, at least partially, be a function of between-group differences in mean. Therefore, a meta-analysis of relative variability of patient compared with control immune parameters scaled to group means was performed: the log coefficient of variation ratio (CVR) (the natural logarithm of the ratio of estimates of population coefficients of variation).^28,86^ Where the mean is greater in patients than controls, the CVR is a more conservative estimate of variability. To aid interpretation, summary effect sizes for lnVR and lnCVR were transformed back to a linear scale, as previously described.^28^ Thus, a VR (or CVR) of 1 indicates equal variability in patient and control groups, a VR (or CVR) greater than 1 indicates greater relative variability in patient groups, and a VR (or CVR) less than 1 indicates lower relative variability in patient groups.

### Meta-analysis of Mean Differences

A meta-analysis of between group differences in immune parameters was performed, indexed using Hedges *g*. A random effects model was used owing to expectation of heterogeneity across studies. Data were log-transformed before meta-analysis, since the Cochrane Collaboration recommends log transformation for normalization of positive skew.^58^ Log-transformed data were either extracted directly from manuscripts, or following provision of raw study-level data by authors which we subsequently log-transformed. Where log-transformed study level/summary data were unavailable, log-transformation estimates were calculated as described by Higgins and colleagues.^61^

### Sensitivity Analyses

To determine if findings were influenced by confounding, we aimed to perform sensitivity analyses to determine if findings remained in studies that matched patients and controls for BMI, smoking, age, gender, ethnicity, and HPA axis activity. We also performed sensitivity analyses excluding poor quality studies.

To determine if findings were influenced by data-skew, we conducted sensitivity analyses after excluding data sets that met Cochrane criteria for skew despite log transformation.^61,88^ The skew ratio of each immune parameter for patients and controls was determined using the following calculation: lowest possible value for each parameter subtracted from observed mean, divided by standard deviation. A ratio of <1 provides strong evidence of skew,^61,88^ and, consequently, studies with a ratio <1 were removed in sensitivity analyses.

To determine if there was a difference in proportion of skewed data between patients and controls (which could influence variability analyses), for each immune parameter, the proportion of data sets with severe skew in patients and controls was compared using Fisher’s exact test.^89^

### Consideration of Publication Bias, Study Inconsistency, and Study Quality

Publication bias was assessed for mean differences in all parameters by visual inspection of funnel plots of standard errors against immune residuals. We did not assess for publication bias related to variability, as a selective publication bias is extremely unlikely to exist for such measures. Study quality was assessed using the Newcastle-Ottawa Scale (NOS) (supplementary eAppendix 4).^61,90^ The thresholds for converting NOS scores into “good,” “fair,” and “poor” quality followed criteria previously described by systematic reviews^91^ sponsored by the US Agency for Healthcare Research and Quality (supplementary eAppendix 4). Analyses were repeated with poor quality studies removed. Inconsistency between studies was assessed using the *I*^2^ statistic (supplementary eAppendix 3).^92^

### Consideration of Raw Data Distribution

For patients and controls, distribution of raw data sets (with >10 data points) for each immune parameter was visually examined using kernel density plots. Studies providing data for the same immune parameter were examined on the same kernel density plot, with values first normalized (mean-scaled). Hartigan’s Dip Test of Unimodality^93^ was employed to assess the probability of immune parameter data following a unimodal distribution.

## Results

### Study Selection

Of 3905 citations retrieved, 3751 were excluded after title/abstract review (supplementary eFigure 1). Following manuscript review, 115 studies were excluded based on failure to meet inclusion criteria. All studies included were cross-sectional. IL2 and IL10 data for patients and controls from 2 data sets^54–57^ were excluded owing to insufficient data to allow mean/standard deviation calculation. The final data set included 35 publications,^26,40–60,69–82^ making up 32 study data sets providing data on 188 immune measures (number of measures is greater than number of data sets because subjects had more than one measure in many studies). The total sample consisted of 2733 people (1263 patients, 1470 controls; Table 1, supplementary eFigure 1 and supplementary eTable 1), allowing meta-analysis of IL1β, IL2, sIL2R, IL4, IL6, IL8, IL10, IL17, TNFα, IFNγ, TGFβ, CRP, and total lymphocyte count. We received responses from authors of 15 studies, either providing raw data or clarification regarding data modal distribution/medication status.^26,40–43,45,49–52,54–57,74^ Raw data were obtained for 13 studies.^26,41–43,49–52,54–57,74^ After exclusion of data with <10 data points,^26,43,50^ there were sufficient data to separately analyze the distribution of 65 immune measures (32 in patients, 33 in controls) pertaining to IL2, IL4, IL6, IL8, IL10, TGFβ, TNFα, IFNγ, and CRP. The total sample for raw data analysis was 691 (389 patients, 302 controls).

### Variability Ratio

**Figure 1.**
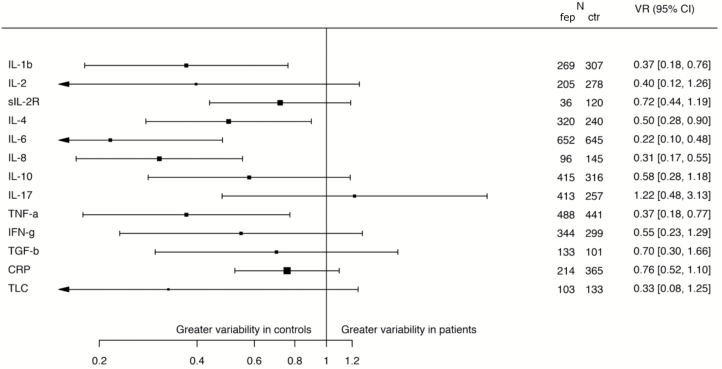
Forest plot showing effect sizes for variability ratio (VR) of immune parameters in antipsychotic-naïve first-episode psychosis compared with healthy controls. The VR was significantly decreased for interleukin 4 (IL4), interleukin 1beta (IL1β), interleukin 6 (IL6), interleukin 8 (IL8), and tumor necrosis factor alpha (TNFα), indicating lower variability of these immune parameters in patients compared with controls.

We found a significant overall effect of group on log variability ratio across all immune parameters (χ^2^ = 150.33, *P* < .0001). Figure 1 shows that the variability of FEP was significantly reduced compared with controls for the following parameters: IL1β (VR = 0.35; 95% CI = 0.17–0.72; *P* = .004); IL6 (VR = 0.19; 95% CI = 0.09–0.43; *P* < .0001); IL8 (VR = 0.28; 95% CI = 0.15–0.52; *P* < .0001); TNFα (VR = 0.36; 95% CI = 0.17–0.75; *P =* .01); and IL4 (VR = 0.55; 95% CI = 0.32–0.94; *P =* .03). Variability was not significantly altered for: IL17 (VR = 0.61; 95% CI = 0.35–1.08; *P =* .09); CRP (VR = 0.82; 95% CI = 0.45–1.47; *P =* .50); IFNγ (VR = 0.62; 95% CI = 0.25–1.50; *P =* .29); IL10 (VR = 0.58; 95% CI = 0.27–1.21; *P =* .15); IL2 (VR = 0.31; 95% CI = 0.09–1.13; *P =* .08); TGFβ (VR = 0.99; 95% CI = 0.59–1.65; *P =* .97); sIL2R (VR = 0.79; 95% CI = 0.48–1.32; *P =* .38); and total lymphocyte cell count (VR = 0.33; 95% CI = 0.08–1.25; *P =* .10).

### Coefficient of Variation Ratio

**Figure 2.**
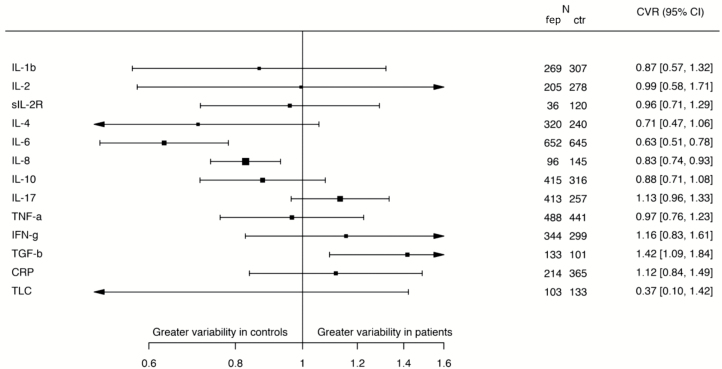
Forest plot showing effect sizes for mean-scaled variability of immune parameters in antipsychotic-naïve first-episode psychosis compared with healthy controls. The coefficient of variation ratio (CVR) was significant decreased for interleukin 6 (IL6) and interleukin 8 (IL8), indicating lower variability of these immune parameters in patients compared with controls, and significantly increased for transforming growth factor beta (TGFβ), indicating increased variability of this immune parameter in patients compared with controls.

We found a significant overall effect of group on log variability ratio across all immune parameters (χ^2^ = 68.62, *P* < .0001). Figure 2 shows that significant variability differences found with VR remained present using CVR for IL6 (CVR = 0.64; 95% CI = 0.52–0.79; *P <* .0001) and IL8 (CVR = 0.83; 95% CI = 0.74–0.93; *P* = .001). There was no significant difference found in variability of CRP (CVR = 1.12; 95% CI = 0.84–1.49; *P =* .46), IFNγ (CVR = 1.16; 95% CI = 0.83–1.62; *P =* .39), IL10 (CVR = 0.88; 95% CI = 0.71–1.08; *P =* .23), IL2 (CVR = 1.00; 95% CI = 0.58–1.73; *P =* .99), IL17 (CVR = 1.13; 95% CI = 0.96–1.33; *P =* .13), and total lymphocyte count (CVR = 0.37; 95% CI = 0.10–1.42; *P =* .15), consistent with VR results. However, differences in variability for IL1β (CVR = 0.87; 95% CI = 0.57–1.33; *P =* .52), IL4 (CVR = 0.71; 95% CI = 0.48–1.07; *P =* .10), and TNFα (CVR = 0.97; 95% CI = 0.76–1.23; *P =* .79), shown to be significantly less variable in patients as per VR results, were not significant using CVR. Moreover, variability of TGFβ, previously shown in VR analysis to be no different between groups, was more variable in patients according to CVR analysis (CVR = 1.41; 95% CI = 1.09–1.83; *P =* .01). This suggests IL1β, TNFα, IL4, and TGFβ variability analyses are influenced by mean scaling.

### Mean Differences in Immune Measures

**Figure 3.**
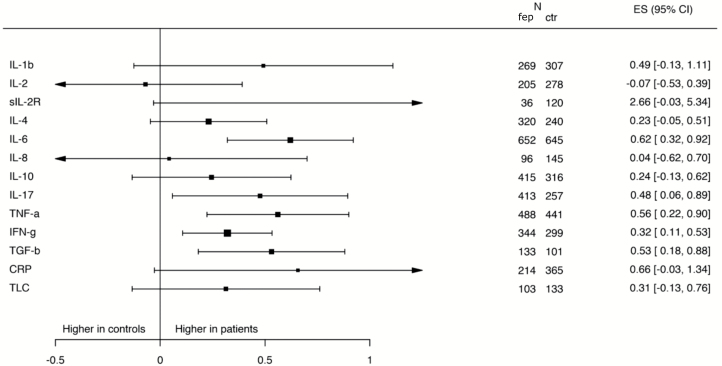
Forest plot showing effect sizes for mean differences in immune parameters in antipsychotic-naïve first-episode psychosis compared with healthy controls. There was a significant elevation in interferon gamma (IFNγ), interleukin 17 (IL17), interleukin 6 (IL6), transforming growth factor beta (TGFβ), and tumor necrosis factor alpha (TNFα) in patients compared with controls. There was no significant difference in C-reactive protein (CRP), interleukin 10 (IL10), interleukin 1beta (IL1β), interleukin 2 (IL2), interleukin 4 (IL4), interleukin 8 (IL8), soluble interleukin 2 receptor (sIL2R), and total lymphocyte count (TLC) in patients compared with controls.

We found a significant overall effect of group on mean levels across all immune parameters (χ^2^ = 114.49, *P* < .0001). Figure 3 shows that significant elevations in the following parameters were observed in FEP: IFNγ (*g* = 0.32; 95% CI = 0.11–0.53; *P* = .003); IL17 (*g* = 0.48; 95% CI = 0.06–0.89; *P* = .03); IL6 (*g* = 0.62; 95% CI = 0.32–0.92; *P <* .0001); TGFβ (*g* = 0.53; 95% CI = 0.18–0.88; *P* = .003); and TNFα (*g* = 0.56; 95% CI = 0.22–0.90; *P* = .001). There were no significant differences between groups for: CRP (*g* = 0.66; 95% CI = −0.03 to 1.34; *P =* .06); total lymphocyte count (*g* = 0.31; 95% CI = −0.13 to 0.76; *P =* .17); IL10 (*g* = 0.24; 95% CI = −0.13 to 0.62; *p =* .20); IL1β (*g* = 0.49; 95% CI = −0.13 to 1.11; *P =* .12); IL2 (*g* = −0.07; 95% CI = −0.53 to 0.39; *P =* .77); IL4 (*g* = 0.23; 95% CI = −0.05 to 0.51; *P =* .10); IL8 (*g* = 0.04; 95% CI = −0.62 to 0.70; *P =* .90); and sIL2R (*g* = 2.66; 95% CI = −0.03 to 5.34; *P =* .05).

### Sensitivity Analysis of the Influence of Confounders

Restricting analysis of mean differences to studies that matched for age, gender, BMI, and smoking created a data set of 8 studies, covering data on 44 immune measures (supplementary eFigure 2). Analysis showed elevated IL6 (*g* = 0.83; 95% CI = 0.21–1.45; *P* = .01), IL17 (*g* = 0.68; 95% CI = 0.09–1.28; *P =* .02), and IFNγ (*g* = 0.54; 95% CI = 0.27–0.80; *P <* .001) in patients, and no significant difference in TNFα, IL10, or IL1β levels between groups. These findings are consistent with the primary meta-analysis, apart from the TNFα outcome, which was no longer significant, suggesting it could be influenced by confounding. There were insufficient data to analyze IL2, sIL2R, IL4, IL8, TGFβ, CRP, and lymphocyte levels. Sensitivity analyses matching for ethnicity and stress were not possible owing to insufficient studies. We combined CRP and hsCRP data a priori (supplementary eAppendix 3). Poor sensitivity of the CRP assay could conceivably mask hsCRP outcomes. Re-analysis of hsCRP data alone suggests this is not the case, with no difference in hsCRP levels between patients and controls (*g* = 0.87; 95% CI = 0.17–1.91; *P =* .10).

### Sensitivity Analysis of the Influence of Residual Skew

Removing studies with evidence of persistent skew despite log transformation created a data set of 27 studies, covering data on 118 immune measures (supplementary eFigure 3). Analysis showed elevated IL6 (*g* = 0.77; 95% CI = 0.15–1.40; *P* = .02), TNFα (*g* = 0.58; 95% CI = 0.08–1.09; *P =* .02), TGFβ (*g* = 0.68; 95% CI = 0.28–1.08; *P* < .01), IFNγ (*g* = 0.24; 95% CI = 0.01–0.48; *P =* .04), and IL17 (*g* = 0.64; 95% CI = 0.15–1.14; *P =* .01) in patients, and no significant difference in IL2, IL4, IL10, CRP, or total lymphocyte count between groups, consistent with our primary meta-analysis. In contrast to the primary analysis, IL1β (*g* = 0.96; 95% CI = 0.39–1.52; *P <* .01), sIL2R (*g* = 0.93; 95% CI = 0.14–1.71; *P =* .02), and IL8 (*g* = 0.87; 95% CI = 0.24–1.49; *P =* .01) were elevated in patients.

For all immune parameters, there was no significant difference in the proportion of immune measures with severe skew in patients compared with controls, either in raw-scaled (*P* = .20–.99) or log-transformed data sets (*P* = .21–.99) (supplementary eTable 2).

### Study Quality

Newcastle Ottawa Scale quality scores ranged from 0 to 8 (supplementary eTable 3). Of 32 samples, 24 were rated as “good-quality,” 4 as “fair-quality,” and 4 as “poor-quality.”^50,53,58,60^ Of 3 studies examining total lymphocyte counts, 2 were poor quality.^53,58^ Excluding the poor-quality paper^50^ from CRP meta-analysis did not alter outcomes for meta-analysis of mean difference (*g* = 0.76; 95% CI = −0.01 to 1.53; *P =* .05). Excluding this study from variability analyses for CRP showed reduced variability in patients (VR = 0.60; 95% CI = 0.45–0.80; *P =* .001), although there was no difference when mean-scaled (CVR = 1.09; 95% CI = 0.79–1.50; *P =* .61). Excluding the poor-quality paper^60^ from sIL2R meta-analysis did not significantly alter outcomes for meta-analysis of mean-difference (*g* = 3.28; 95% CI = 0.06–6.50; *P =* .05) nor variability analyses.

### Publication Bias and Study Inconsistency

The funnel plot for publication bias did not show evidence of asymmetry (supplementary eFigure 4).^26^ Higgins’ *I*^2^ inconsistency values (supplementary eTable 4) demonstrated a medium-large degree of inconsistency for all parameters, apart from low levels of inconsistency for IFNγ.

### Distribution of Raw Immune Parameter Data

Visual inspection of kernel density plots suggested right skewed unimodal distribution for all immune measures in FEP and controls (supplementary eFigure 5). For patients, 30 of 32 immune measures (94%) met Hartigan’s Dip Test criteria for unimodal distribution (supplementary eTable 5), including data sets for IL2, IL4, IL6, IL8, IL10, TGFβ, TNFα, IFNγ, and CRP. For healthy controls, 33 of 33 immune measures (100%) met Hartigan’s Dip Test criteria for unimodal distribution (supplementary eTable 5), including data sets for IL2, IL4, IL6, IL8, IL10, TGFβ, TNFα, IFNγ, and CRP. In the patient group, 1 of the 6 data sets examining IL6 met criteria for a multimodal distribution,^54–56^ and 1 of the 3 data sets examining IFNγ met criteria for a multimodal distribution.^49,74^ These 2 data sets did not have overlapping samples and multimodality for IL6 was not accompanied by multimodality for IFNγ (supplementary eTable 5).

## Discussion

### Summary of Findings

Our first main finding is that that there is a significant reduction in variability of IL1β, IL4, IL6, IL8, and TNFα in FEP patients compared with controls, which is not explained by mean scaling for IL6 and IL8. After adjusting for mean scaling, there was increased heterogeneity of TGFβ in patients compared with controls. As there is no significant difference in proportion of studies with strong evidence of skewed data in patients compared with controls for all parameters, these variability outcomes do not appear to be the result of reduced data skew in patients, and thus may reflect intrinsic differences in immune variability. An examination of raw data did not provide strong evidence for multimodal data distribution of immune parameters in either patients or controls.

We found elevated IL6, TNFα, IFNγ, TGFβ, and IL17 levels in patients compared with controls with small-medium effect sizes (range: *g* = 0.32–0.62). IL6, IFNγ, and IL17 outcomes were robust to sensitivity analyses, indicating these alterations are unlikely to be driven by key potential confounders and data skew.

The absence of variability elevations of most immune parameters in patients compared to controls and the absence of multimodal distribution of most data is evidence against the existence of an immune subgroup of psychosis. Lower variability of IL6 in patients, coupled with a robust difference in mean concentration, could instead be interpreted as this parameter representing a core (or at least more uniformly present) component in the immunobiology of psychosis.

### Strengths and Limitations

By focusing on antipsychotic-naïve FEP, we limited duration of secondary illness-related factors known to influence immune parameters, eg, antipsychotics,^67^ poor diet, and reduced exercise levels.^30,31^ Furthermore, sensitivity analyses focusing on studies with strict environmental and physiological matching provides greater confidence that FEP is associated with elevated immune parameters. Relative to previous meta-analyses in the field, log-transformation to reduce influence of skew on summary effect sizes followed by sensitivity analysis excluding data with persistent evidence of skew provides us with robust evidence that immune alterations are present even when influence of data skew is reduced. Moreover, use of a multivariate meta-analytic approach that models the covariance of immune parameters and allows omnibus testing of results thereby reducing multiplicity concerns, is a strength. Gaining access to raw data to examine distribution of multiple data sets is a further strength, complementing findings of the variability meta-analysis. Finally, since in biological systems variance often scales to mean,^87^ performing a mean-scaled (CVR) variability meta-analysis to complement the primary meta-analysis of variability (VR) provides a conservative approach to assess if primary outcomes are influenced by mean group differences, and provides greater confidence regarding the outcome of increased homogeneity of IL6 and IL8 in patients compared with controls.

It should be noted that there is diagnostic heterogeneity in first-episode samples. In general, about two-thirds of FEP patients have a diagnosis of schizophrenia, while the remainder are diagnosed with other psychotic disorders.^94^ Of the 32 samples, 18 (56%) included patients with schizophrenia alone (table 1). The remaining samples potentially also included affective psychosis. The proportion of individuals with nonaffective psychosis was not defined, precluding us conducting sensitivity analyses of the effect of diagnosis. Therefore, our findings should not be taken as specific to schizophrenia, but representative of FEP in general. Future studies should provide greater clarity as to whether patients included in analyses presented with an affective or nonaffective psychosis. The combination of affective and nonaffective psychosis in variability analyses should increase heterogeneity in patients and might be expected to influence the modal distribution of patient immune data. However, there was reduced variability of several immune parameters in patients, and no strong evidence for multimodal distribution of patient immune data. Indeed, inclusion of affective psychosis within patient samples may have resulted in under-estimation of increased homogeneity of immune variability in this group, compared with controls.

Inconsistency between studies was moderate to high. This could reflect methodological factors, eg, differences in assay sensitivity, and use of serum/plasma sampling. However, the random effects model used is robust to inconsistency, and would not explain our variability findings, because these reflect within-study variation (where methodologic factors are the same in both patient and control groups in any given study). Moreover, although we were stringent in selecting antipsychotic naïve patients, confirmation of naivety for all psychiatric medications was not universally stated. Thus, use of treatments beyond antipsychotics may have confounded results contributing to inconsistency. Future prospective studies are required that control stringently for medication thereby addressing this potential confound.

Although sample sizes are larger than previous meta-analyses, relatively small sample sizes persist for measures of sIL2R (*n* = 156), TGFβ (*n* = 234), and lymphocytes (*n* = 236). Thus, conclusions regarding these parameters are less secure, and further studies required. Furthermore, only 8 out of 32 (25%) studies matched simultaneously for age, gender, smoking, and BMI, and 2 sensitivity analyses, matching for ethnicity and stress, were not possible owing to insufficient studies. Other factors that could be different between the groups and influence immune measures include recreational substance exposure^95^ and subclinical physical comorbidity.^96–98^ Information on these was not included in most studies, precluding sensitivity analysis. Future studies should aim to match patients appropriately to reduce impact of these potential confounds. Moreover, controls may be unusually healthy compared with the general population because they are screened for illnesses, potentially inflating effect sizes for mean differences.^99^ However, inclusion of super-healthy controls would reduce variability of control immune parameters. In fact, we observed the opposite, and the inclusion of “super controls” could even have led to an under-estimate of reduced variability in immune measures in patients. There is the potential for variability analyses to have been influenced by the presence of latent immune subgroups within the control population. We are however reassured by our findings that 100% of raw control data examined met criteria for unimodal distribution.

Although all studies included in analyses used well-validated quantification techniques (supplementary eTable 6), insufficient assay sensitivity may have limited ability to detect subtle differences in immune parameters between groups, particularly for titers beneath the limit of assay detection. However, where we could assess impact of poor assay sensitivity, examining hsCRP levels separate from CRP levels, results were unaltered. Future developments in immunoassay technology which bring greater assay sensitivity^100^ will potentially provide greater clarity as to the nature of immune alterations in psychosis.

Positive data skew can inflate standard deviation owing to the presence of outliers within the “tail” of the data.^101^ We observed evidence of data skew in patients and/or controls for all immune parameters except IL8, TGFβ, and lymphocyte count (supplementary eTable 2). For remaining immune parameters, residual data skew may have influenced variability analyses. However, we demonstrated no significant difference in proportion of skewed data between groups, suggesting that differences in skew in controls compared with patients do not explain variability differences. Moreover, skew does not always inflate standard deviations, since skew describes the shape of data distribution, not scale of spread.^94^

### Comparison With Previous Meta-analyses

A summary of results from previous meta-analyses examining mean differences in immune parameters in FEP^16–22^ is provided in supplementary eTable 7. There are 2 key differences when compared with previous meta-analyses. First, we failed to observe any differences between patients and controls for mean levels of IL1β, sIL2R, IL4, IL8, IL10, total lymphocyte count, and CRP. However, IL1β, sIL2R, and IL8 levels were elevated in sensitivity analyses excluding data with evidence of skew. These sensitivity analyses are limited by reduced sample size, but suggest the need for future research to determine whether differences in these immune parameters exist between FEP and controls. Second, where differences were observed, effect size estimates were generally smaller than those in previous meta-analyses. For example, Upthegrove et al^19^ previously reported effect sizes of 2.21 and 0.94 for elevated IL6 and TNFα, respectively in antipsychotic-naïve FEP compared with controls, whereas we observed effect sizes of 0.62 and 0.56, respectively. These differences may be a consequence of several factors, including increased sample size (participant numbers increasing by up to 7-fold), use of log transformed data, and a multivariate meta-analytic approach that models covariance of immune parameters. Given the additional sample size, the focus on antipsychotic naïve patients, the statistical approach employed, and steps we have taken to assess the influence of data-skew and physiological confounds, we suggest that our updated results are likely the most reliable estimates of peripheral immune alterations in FEP to date.

### Interpretation and Implications

Our findings suggest that an immune subtype of psychosis, if present, cannot currently be identified through examination of peripheral immune parameter distribution. Indeed, our results could instead be interpreted as supporting the hypothesis that alterations in the immune system are a general feature of psychosis.^102^

Our results cannot however exclude the possibility that there are alterations in other aspects of the immune system specific to a subgroup of patients. An immune subgroup could manifest itself via various peripheral patterns. Firstly, an immune subgroup might result in wider spread of data points (increased heterogeneity) in patients compared with healthy controls, although, with the potential exception of TGFβ, this is not observed in the current meta-analysis. Secondly, subgroups of patients defined by step increases in immune parameter concentrations could result in multimodal distribution of immune parameter data. Modal analyses in this article suggest a unimodal distribution for most immune parameters, although there was some evidence, albeit weak, for a multimodal distribution of IL6 and IFNγ. Thirdly, a proportion of patients may be more vulnerable to the impact of immune activation, inducing inflammatory and thence psychopathological sequelae in that group (even if proinflammatory titers are of a similar magnitude compared with controls). The observation of increased variability of TGFβ levels in FEP compared with controls following mean scaling could reflect alterations in immune regulatory pathways in a subgroup of individuals with FEP, supporting this model. TGFβ can (although not always)^103^ perform an anti-inflammatory role, including inhibition of cytokine production from macrophages^104^ and inhibition of B-lymphocyte proliferation.^105^ Thus, patients with an impaired TGFβ-mediated immune response could potentially be vulnerable to proinflammatory effects that characterize psychosis (ie, homogenous increases in IL6). Moreover, genetic variants in the TGFβ gene TGFB1 influence susceptibility for schizophrenia.^106^ Fourthly, immune susceptibility may arise through a unique network-effect of multiple cytokines to bring about inflammatory sequelae and thence psychopathology. Indeed, Weickert and colleagues have employed cluster analysis to divide patients with schizophrenia-spectrum disorders into subgroups based on elevation of multiple cytokines in unison.^27,29^ They have observed that, compared with controls, a greater proportion of patients clustered within an “elevated cytokine subgroup.” Jeffries and colleagues^107^ have used graph theory to examine network connectivity of blood proteins related to neuroimmunology across the psychosis spectrum, observing that protein correlation networks can successfully differentiate between controls, and prodromal individuals who transition/do not transition to psychosis. Based on the current meta-analysis, we are unable to comment on whether there is a FEP immune subgroup characterized by an abnormal immune parameter network.

The findings of elevated IL6, IL17, and IFNγ in psychosis appear robust, as elevations are also seen in sensitivity analyses, indicating they are unlikely due to confounding or other nonspecific factors. Elevations of these 3 cytokines could point toward activation of both innate and adaptive immune responses.^108,109^ Moreover, of these 3 cytokines, IL6 shows reduced variability in patients (including after mean scaling), suggesting this could be a core component of the pathophysiology of psychosis. IL6 is a multifunctional cytokine playing a role in inflammation and the acute phase response,^110^ immune response,^111–113^ hematopoiesis,^114^ glucose and lipid metabolism,^115^ and bone-turnover.^116^ It crosses the blood–brain barrier (BBB),^117^ and there is evidence of increased cerebrospinal fluid (CSF) IL6 levels in psychosis,^13^ indicating that peripheral IL6 could influence brain function. In the CNS, IL6 induces microglial proliferation^118^ and activation.^119^ Microglia play a key role in synaptic remodeling, among other functions.^120^ Thus, raised peripheral IL6 levels could activate microglia and influence secondary brain changes.^4^ Supporting this, postmortem studies have reported increased microglial density and morphological changes indicative of microglial activation in schizophrenia,^8–10^ and some, although not all, positron emission tomography in vivo studies have reported altered binding to a marker expressed on activated microglia in schizophrenia and people at risk of psychosis.^11,12^ However, it is important to recognize that there are other potential models explaining the association between immune alterations and psychosis,^98^ and recent studies examining therapeutic potential of anti-IL6 immunotherapy in schizophrenia have been disappointing (although this potentially relates to the inability of the monoclonal antibody to cross the BBB).^121,122^ Immune alterations could be a consequence of psychosis (eg, psychosocial stress activating an inflammatory response^123^) or simply an epiphenomenon. Thus, whether peripheral alterations are a cause or a consequence of psychosis remain to be determined.^4^ Longitudinal studies examining networks of immune parameters in both CSF and blood following individuals in the prodrome through transition to FEP and experimental medicine studies modulating specific aspects of immune function are needed to identify the mechanism underlying the immune alterations we report, and determine the potential for targeting them therapeutically.

## Conclusions

Antipsychotic naïve FEP is associated with elevated levels of IL6, IL17, and IFNγ with small-medium effect sizes after accounting for skew and physiological confounds. There is reduced variability in the levels of immune parameters in FEP, and there does not appear to be a clear multimodal distribution of immune parameters in patients. These findings suggest that an immune subgroup of psychosis cannot currently be defined by examination of peripheral immune data spread, and are consistent with elevated immune markers being typical of psychosis.

**Summary**

1. Antipsychotic-naïve patients with first-episode psychosis show elevated IL6, TNFα, IFNγ, IL17, and TGFβ levels compared with healthy controls.
2. Patients do not show differences in CRP, total lymphocyte counts, IL1β, IL2, sIL2R, IL4, IL8, and IL10 levels compared with healthy controls.
3. Elevated levels of IL6, IL17, and IFNγ are robust to sensitivity analyses which account for data skew, study quality, and physiological/environmental matching.
4. There is reduced variability of immune parameters in patients. Moreover, examination of raw data does not show a multi-modal distribution of immune parameter data in patients.
5. These findings suggest that an immune subgroup of psychosis cannot currently be defined by examination of peripheral immune data spread, and are consistent with elevated immune markers being typical of psychosis.

## Funding

This study was funded by grants MC-A656-5QD30 from the Medical Research Council-UK, 666 from the Maudsley Charity 094849/Z/10/Z from the Brain and Behavior Research Foundation, and Wellcome Trust (Dr Howes) and the National Institute for Health Research Biomedical Research Centre at South London and Maudsley National Health Service Foundation Trust and King’s College London. R.M.’s work is supported by the Wellcome Trust (200102/Z/15/Z). V.M. receives funding support from MQ and from the National Institute for Health Research Mental Health Biomedical Research Centre at South London and Maudsley NHS Foundation Trust and King’s College London.

## Conflict of interest

Dr Howes has received investigator-initiated research funding from and/or participated in advisory/speaker meetings organized by AstraZeneca, Autifony, BMS, Eli Lilly, Heptares, Janssen, Lundbeck, Lyden-Delta, Otsuka, Servier, Sunovion, Rand, and Roche. Drs Pillinger, Osimo, Brugger, Mondelli, and McCutcheon report no conflicts of interest.

## Supplementary Material

sby160_suppl_Supplementary_Material
