# Supplementary material for: A Meta-analysis of Immune Parameters, Variability, and Assessment of Modal Distribution in Psychosis and Test of the Immune Subgroup Hypothesis
Source: Schizophr Bull. 2018 Nov 8;45(5):1120–33. doi: 10.1093/schbul/sby160 (PMC6737479; doi:10.1093/schbul/sby160)
Supplement: sby160_suppl_Supplementary_Material [file sby160_suppl_supplementary_material.docx]

**SUPPLEMENTARY INFORMATION**

**CONTENTS**

Pages 2-5 eAppendix 1: PRISMA and MOOSE checklists

Pages 6-7 eAppendix 2: A priori protocol

Pages 8-9 eAppendix 3: Supplementary methods

Page 10 eAppendix 4: Newcastle Ottawa Scale definitions

Page 11 eFigure 1: Search process

Page 12 eFigure 2: Forest plot for sensitivity analysis with severe data skew excluded

eFigure 3: Forest plot for sensitivity analysis matching for BMI/age/gender/smoking

Page 13 eFigure 4: Multivariate funnel plot

Pages 14-15 eFigure 5: Kernel density plots of raw immune parameter data

Page 16 eTable 1: Raw data used in all meta-analyses

Pages 17-19 eTable 2: Skew ratio comparisons

Page 20 eTable 3: Newcastle Ottawa Scale results

Page 21 eTable 4: Inconsistency assessment using I^2^ values

Pages 22-24 eTable 5: Hartigan dip test for normality assessments of raw immune parameter data

Page 25 eTable 5: Immune parameter measurement technique

Page 26 eTable 6: Comparison with previous meta-analyses

Pages 27-30 Supplementary References

**eAppendix 1**

**PRISMA (2009) CHECKLIST**

| Section/Topic | # | Checklist Item | Reported on page # |
| --- | --- | --- | --- |
| TITLE | | | |
| Title | 1 | Identify the report as a systematic review, meta-analysis, or both. | 1 |
| ABSTRACT | | | |
| Structured summary | 2 | Provide a structured summary including, as applicable: background; objectives; data sources; study eligibility criteria, participants, and interventions; study appraisal and synthesis methods; results; limitations; conclusions and implications of key findings; systematic review registration number. | 2 |
| INTRODUCTION | | | |
| Rationale | 3 | Describe the rationale for the review in the context of what is already known. | 3,4 |
| Objective | 4 | Provide an explicit statement of questions being addressed with reference to participants, interventions, comparisons, outcomes, and study design (PICOS). | 3,4 |
| METHODS | | | |
| Protocol and registration | 5 | Indicate if a review protocol exists, if and where it can be accessed (e.g., Web address), and, if available, provide registration information including registration number. | 4 |
| Eligibility criteria | 6 | Specify study characteristics (e.g., PICOS, length of follow-up) and report characteristics (e.g., years considered, language, publication status) used as criteria for eligibility, giving rationale. | 4,5 |
| Information sources | 7 | Describe all information sources (e.g., databases with dates of coverage, contact with study authors to identify additional studies) in the search and date last searched. | 4 |
| Search | 8 | Present full electronic search strategy for at least one database, including any limits used, such that it could be repeated. | 4 |
| Study selection | 9 | State the process for selecting studies (i.e., screening, eligibility, included in systematic review, and, if applicable, included in the meta-analysis). | 4,5 |
| Data collection process | 10 | Describe method of data extraction from reports (e.g., piloted forms, independently, in duplicate) and any processes for obtaining and confirming data from investigators. | 5 |
| Data items | 11 | List and define all variables for which data were sought (e.g., PICOS, funding sources) and any assumptions and simplifications made. | 5 |
| Risk of bias in individual studies | 12 | Describe methods used for assessing risk of bias of individual studies (including specification of whether this was done at the study or outcome level), and how this information is to be used in any data synthesis. | 7 |
| Summary measures | 13 | State the principal summary measures (e.g., risk ratio, difference in means). | 7,8 |
| Synthesis of results | 14 | Describe the methods of handling data and combining results of studies, if done, including measures of consistency (e.g., I^2^) for each meta-analysis. | 7,8,9 |
| Risk of bias across studies | 15 | Specify any assessment of risk of bias that may affect the cumulative evidence (e.g., publication bias, selective reporting within studies). | 7 |
| Additional analyses | 16 | Describe methods of additional analyses (e.g., sensitivity or subgroup analyses, meta-regression), if done, indicating which were pre-specified. | 7,8 |
| RESULTS | | | |
| Study selection | 17 | Give numbers of studies screened, assessed for eligibility, and included in the review, with reasons for exclusions at each stage, ideally with a flow diagram. | 7 |
| Study characteristics | 18 | For each study, present characteristics for which data were extracted (e.g., study size, PICOS, follow-up period) and provide the citations. | Table 1 |
| Risk of bias within studies | 19 | Present data on risk of bias of each study and, if available, any outcome level assessment (see item 12). | 10 |
| Results of individual studies | 20 | For all outcomes considered (benefits or harms), present, for each study: (a) simple summary data for each intervention group (b) effect estimates and confidence intervals, ideally with a forest plot. | Figures 1-3 |
| Synthesis of results | 21 | Present results of each meta-analysis done, including confidence intervals and measures of consistency. | 7,8,9,10 |
| Risk of bias across studies | 22 | Present results of any assessment of risk of bias across studies (see Item 15). | 10 and eFigure 4 |
| Additional analysis | 23 | Give results of additional analyses, if done (e.g., sensitivity or subgroup analyses, meta-regression [see Item 16]). | 9 |
| DISCUSSION | | | |
| Summary of evidence | 24 | Summarize the main findings including the strength of evidence for each main outcome; consider their relevance to key groups (e.g., healthcare providers, users, and policy makers). | 12 |
| Limitations | 25 | Discuss limitations at study and outcome level (e.g., risk of bias), and at review-level (e.g., incomplete retrieval of identified research, reporting bias). | 12-14 |
| Conclusions | 26 | Provide a general interpretation of the results in the context of other evidence, and implications for future research. | 17 |
| FUNDING | | | |
| Funding | 27 | Describe sources of funding for the systematic review and other support (e.g., supply of data); role of funders for the systematic review. | 15,16 |

**MOOSE CHECKLIST**

| **Criteria** | **Brief description of how the criteria were handled in the meta-analysis** |
| --- | --- |
| **Reporting of background should include** | |
| Problem definition | Abstract: Immune parameters are elevated in psychosis, but it is unclear whether alterations are homogenous across patients or if there is heterogeneity, consistent with the hypothesis that immune alterations are specific to a subgroup of patients. |
| Hypothesis statement | Introduction: Does increased variability of immune parameters exist in psychosis? This can be assessed using meta-analysis of variability, as has previously been used to examine regional brain structural variability in FEP. If immune alterations are only seen in a sub-group of patients, greater immune measure variability in patients would be predicted, reflecting heterogeneity in immune dysregulation. Conversely, if immune alterations are a core component of the pathophysiology of psychosis, this could result in reduced immune variability in patients. To our knowledge, neither of these questions have been addressed by previous meta-analyses.’ |
| Description of study outcomes | **Methods, selection criteria:** ‘Studies assessing blood cytokines/cytokine receptors, C-reactive protein (CRP), and white cell counts.’ |
| Type of exposure or intervention used | n/a |
| Type of study designs used | All study designs were considered |
| Study Population | **Methods, selection criteria**: ‘antipsychotic-naïve first episode psychosis and healthy controls’ |
| **Reporting of search strategy should include** | |
| Qualifications of searchers | Academic qualifications defined on title page. |
| Search strategy, including time period included in the synthesis and key words | Full selection procedures (data bases, time period, and key words) defined in **Methods, Selection Procedures** |
| Databases and registries searched | The Pubmed, EMBASE, and PsycINFO databases |
| Search Software used, name and version | Ovid: <http://ovidsp.ovid.com/> |
| Use of hand searching | n/a |
| List of citations located and those excluded, including justifications | Defined in **Results, Study Selection** |
| Method of addressing articles published in languages other than English | Only studies in English were considered |
| Methods of handling abstracts and unpublished studies | Defined in **Methods,** **Selection Procedures** |
| **Reporting of methods should include** | |
| Description of relevance or appropriateness of studies assembled for assessing the hypothesis to be tested | Described in **Results, Study Selection**, with data available for meta-analysis of levels of IL1β, IL2, sIL2R, IL4, IL6, IL8, IL10, IL17, TNFα, IFNγ, TGFβ, CRP, and total lymphocyte count. |
| Rationale for the selection and coding of data | n/a |
| Assessment of confounding | Sensitivity analyses performed assessing impact of skewed data and environmental/physiological confounds |
| Assessment of study quality | Assessed using the Newcastle Ottawa Scale |
| Assessment of heterogeneity | Assessed using Higgins’ I^2^ |
| Description of statistical methods in sufficient detail to be replicated | Details provided in methods section and supplementary information. Also, references provided which supply relevant calculations to allow variability analyses to be performed. |
| Provision of appropriate tables and graphics | Search described using diagram (supplementary information), forest plots for all analyses provided, data sets described in table, raw data provided in supplementary information. |
| **Reporting of results should include** | |
| Graph summarising individual study estimates and overall estimate | Forest plots for all analyses provided |
| Table giving descriptive information for each study included | Table 1 |
| Results of sensitivity testing | Reported in results section with supplementary figures showing relevant additional forest plots. |
| Indication of statistical uncertainty of findings | Reported throughout results section. |
| **Reporting of discussion should include** | |
| Quantitative assessment of bias | Funnel plot results reflected upon. |
| Justification for Exclusion | Rationale behind exclusion of medication/chronic patients discussed. |
| Assessment of quality of included studies | Results of Newcastle Ottawa Scale discussed. |
| **Reporting of conclusions should involve** | |
| Considerations of alternative explanations for observed results | Discussion of different theories regarding source of immune alterations in psychosis. |
| Generalisation of the conclusions | Commented on with regards our findings being applicable to psychosis in general (and not necessarily just schizophrenia) |
| Guidelines for future research | Suggestions for future research directions provided throughout discussion. |
| Disclosure of funding source | Full financial disclosures provided. |

**eAppendix 2**

**A priori protocol**

**Working title:** Immune parameters in antipsychotic naïve first episode psychosis

**Language:** English

**Type of review:** Systematic review and meta-analysis

**Details of any existing review of the same topic by the same authors:** None

**Collaborators:**

Dr Toby Pillinger ([toby.pillinger@kcl.ac.uk](mailto:toby.pillinger@kcl.ac.uk))

Dr Emanuele Osimo ([e.osimo@lms.mrc.ac.uk](mailto:e.osimo@lms.mrc.ac.uk))

Dr Robert McCutcheon ([robert.mccutcheon@kcl.ac.uk](mailto:robert.mccutcheon@kcl.ac.uk))

Dr Stefan Brugger ([stefpb@gmail.com](mailto:stefpb@gmail.com))

Professor Oliver Howes ([oliver.howes@kcl.ac.uk](mailto:oliver.howes@kcl.ac.uk))

**Institution:** Institute of Psychiatry, Psychology and Neuroscience, King’s College London

**Funding Sources:** MRC-UK, Maudsley Charity, Brain&Behavior Research Foundation, Wellcome Trust.

**Conflicts of Interest:**

Dr Howes has received investigator-initiated research funding from and/or participated in advisory/ speaker meetings organised by Astra-Zeneca, Autifony, BMS, Eli Lilly, Heptares, Janssen, Lundbeck, Lyden-Delta, Otsuka, Servier, Sunovion, Rand and Roche. Neither Professor Howes nor his family have been employed by or have holdings/a financial stake in any biomedical company. Drs Pillinger, Osimo, McCutcheon and Brugger report no financial relationships with commercial interests.

**Review Question:**

1. Do immune parameters differ in antipsychotic naïve first episode psychosis compared with healthy controls?
2. What is the influence of failure to physiologically match patient and control groups on meta-analytic outcomes?
3. Is there a difference in the variability of immune parameters in antipsychotic naïve first episode psychosis compared with healthy controls?

**Searches:**

Pubmed, EMBASE, and PsycINFO databases. Keywords: (lymphocytes or T-lymphocytes or B-lymphocytes or monocytes or macrophages or inflammat* or IL-* or cytokine or CRP or C-reactive protein or hs-CRP or hsCRP or interleukin* or tumour necrosis factor or transforming growth factor or interferon) and (schizophren* or psycho*) and (first episode or early or antipsychotic* or drug* or neuroleptic*)

**Condition being studied:** Antipsychotic naïve first episode psychosis

**Participants/Population, and Comparator/Control:** Antipsychotic naïve first episode psychosis, healthy controls.

**Primary Outcomes:**

Effect sizes of immune parameter alterations in FEP compared with healthy controls

Consideration of impact of physiological confounders (e.g. BMI)

Variability analysis of immune parameter alterations in FEP compared with healthy controls

**Data Extraction:**

Screening based on title and abstract will be performed independently by two authors (TP and EO). Data extraction from case-control studies referenced in selected meta-analyses will be performed independently (by TP and EO), and any disagreements resolved by rechecking original articles.

**Risk of Bias Assessment:**

Publication bias will assessed be by visual inspection of funnel plots. Heterogeneity will be assessed using the I^2^ statistic. Study quality will be assessed using the Newcastle Ottowa Scale.

**Strategy for Data Synthesis:**

1. A multivariate meta-analysis using an unstructured covariance matrix of between group differences in immune parameters using log transformed data.
2. Sensitivity analyses to examine effect of skew and matching (e.g. BMI) between patient and controls groups on effect size for immune alterations between patients and controls.
3. A meta-analysis of relative variability of patient compared with control immune parameters, and a meta-analysis of relative variability of patient compared with control immune parameters scaled to group means.

All statistical analyses will be conducted using the metafor package in the R statistical programming language.

**eAppendix 3**

**Supplementary Methods**

**Selection Criteria**

Inclusion criteria were: 1) Patients with FEP, defined either as first treatment contact or patients recruited from FEP services in line with previous studies;^53^ 2) Antipsychotic-naïve; 3) A healthy control group; 4) Studies assessing blood cytokines/cytokine receptors, C-reactive protein (CRP), and white cell counts. The rationale behind focussing on antipsychotic-naïve FEP was to minimise confounding effects of medication and lifestyle habits (e.g. diet and exercise) associated with chronic psychotic illness^54, 55^ that may directly (e.g. antipsychotics)^56^ or indirectly (e.g. diet/exercise)^57, 58^ alter immune parameters. Exclusion criteria were: 1) Genetic studies; 2) *In vitro* studies; 3) Studies looking at stimulated levels of cytokines as they reflect the consequences of immune challenge as opposed to basal immune activity; 4) Substance or medication induced psychotic disorder; 5) Absence of data allowing mean and/or standard deviation to be determined. Where insufficient data were recorded for a given immune parameter to allow for mean and/or standard deviation to be calculated in either the patient or control group, data pertaining to that parameter was excluded for both the patient and control group for that study.

**Recorded variables**

Data were extracted according to the following model: author, year of publication, participant matching criteria: age, gender, body mass index, smoking status, ethnicity, blood cortisol levels, duration of psychosis, confirmation of antipsychotic-naivety, and mean (with standard deviation) measure of immune parameter (Table 1 and eTable 2). Further details regarding recorded variables are provided in eAppendix 3.

Where data were available only in diagrammatic format, data were extracted using the Plot Digitizer tool (<http://plotdigitizer.sourceforge.net/>). Where there were multiple publications for the same data set, authors were contacted to clarify which data sets pertained uniquely to which study. In case of failure of response from authors, data were extracted only from the study with the largest data set. Authors were also contacted to obtain entirely antipsychotic naïve data sets. Similarly, where the report did not confirm that data were normally distributed, authors were contacted and study-level data requested.

**Combination of data sets for meta-analysis**

CRP and hsCRP data sets were combined for analyses since both analyses measure CRP levels, only using different assays.^1^ Studies reporting IL17 and IL17A were combined respectively for analyses, since IL17 is homologous to IL17A.^2^ The sIL2R is made up of three subunit chains: alpha; beta; and gamma. Unless specified in the methods of a paper that a study was examining sILR beta or gamma chains, or on examination of the immunoassay employed by the authors that beta or gamma chains of the receptor were being quantified, sIL2R quantification was assumed to be measuring sIL2R alpha (sILR2A) chains and as such were combined with sIL2R for analysis. The rationale behind this is that the alpha chain forms the majority of the sIL2R found in serum,^3^ and among the broader literature, studies examining sIL2R within the periphery are in fact measuring levels of sIL2RA.^4^

**Meta-analysis of variability**

To test the hypothesis of increased variability in immune parameters in psychosis, we performed a meta-analysis of relative variability of immune parameters in patients compared with controls. This analysis used the dataset described above. The variability analysis includes log transformation to normalise the sampling distribution of the standard deviation.

**Consideration of publication bias, study inconsistency, and study quality**

Inconsistency was assessed using the I^2^ statistic, with an I^2^ of less than 25% deemed to have low inconsistency, 25-75% medium inconsistency, and greater than 75% high inconsistency.^75^

**eAppendix 4**

**Newcastle Ottawa Quality assessment scale for case control studies**

**SELECTION**

**1) Is the case definition adequate?**

*a) yes, with independent validation (1 point)*

b) yes, e.g. record linkage or based on self-reports

c) no description

**2) Representativeness of the cases**

*a) consecutive or obviously representative series of cases (1 point)*

b) potential for selection biases or not stated

**3) Selection of Controls**

*a) community controls (1 point)*

b) hospital controls

c) no description

**4) Definition of Controls**

*a) no history of disease (1 point)*

b) no description of source

**COMPARABILITY**

**1) Comparability of cases and controls based on the design or analysis**

*a) study controls for age (1 point)*

*b) study controls for BMI (1 point)*

**EXPOSURE**

**1) Ascertainment of exposure**

*a) secure record (1 point)*

*b) structured interview where blind to case/control status (1 point)*

c) interview not blinded to case/control status

d) written self-report or medical record only

e) no description

**2) Same method of ascertainment for cases and controls**

*a) yes (1 point)*

b) no

**eFigure 1**

**Search process**

**
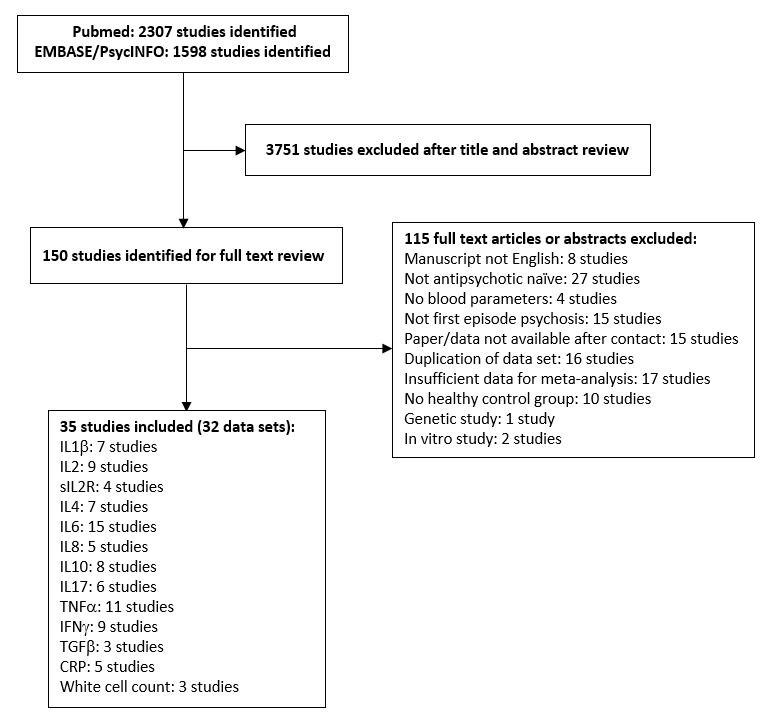
**

**eFigure 2**

**Forest plot showing effect sizes for mean differences in log transformed immune parameters in antipsychotic naïve first episode psychosis compared with healthy controls – BMI, age, gender and smoking-matched dataset.**


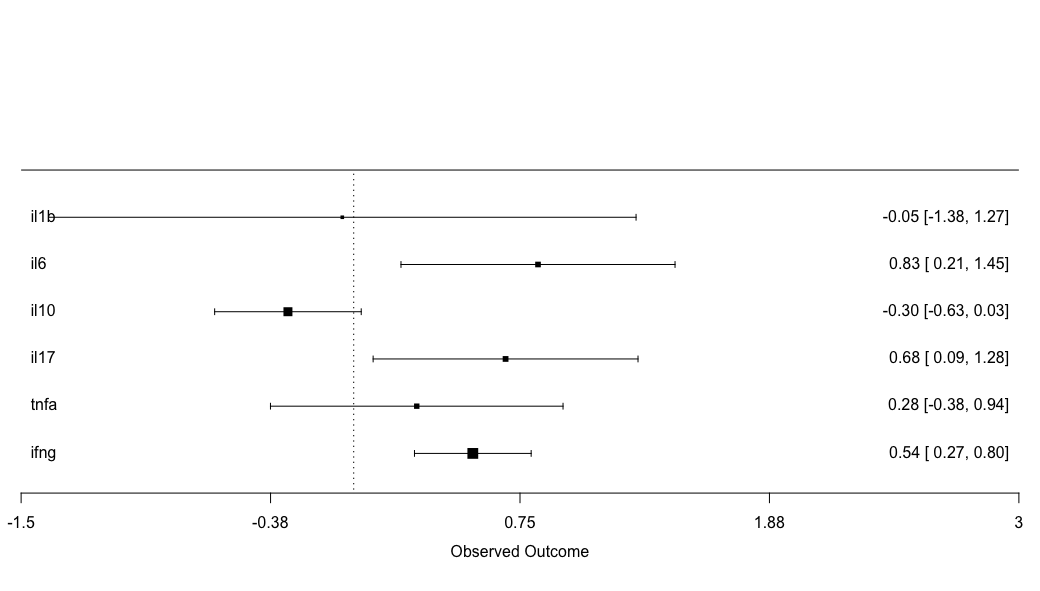


**eFigure 3**

**Forest plot showing effect sizes for mean differences in log transformed immune parameters in antipsychotic naïve first episode psychosis compared with healthy controls – non-skewed dataset.**

**
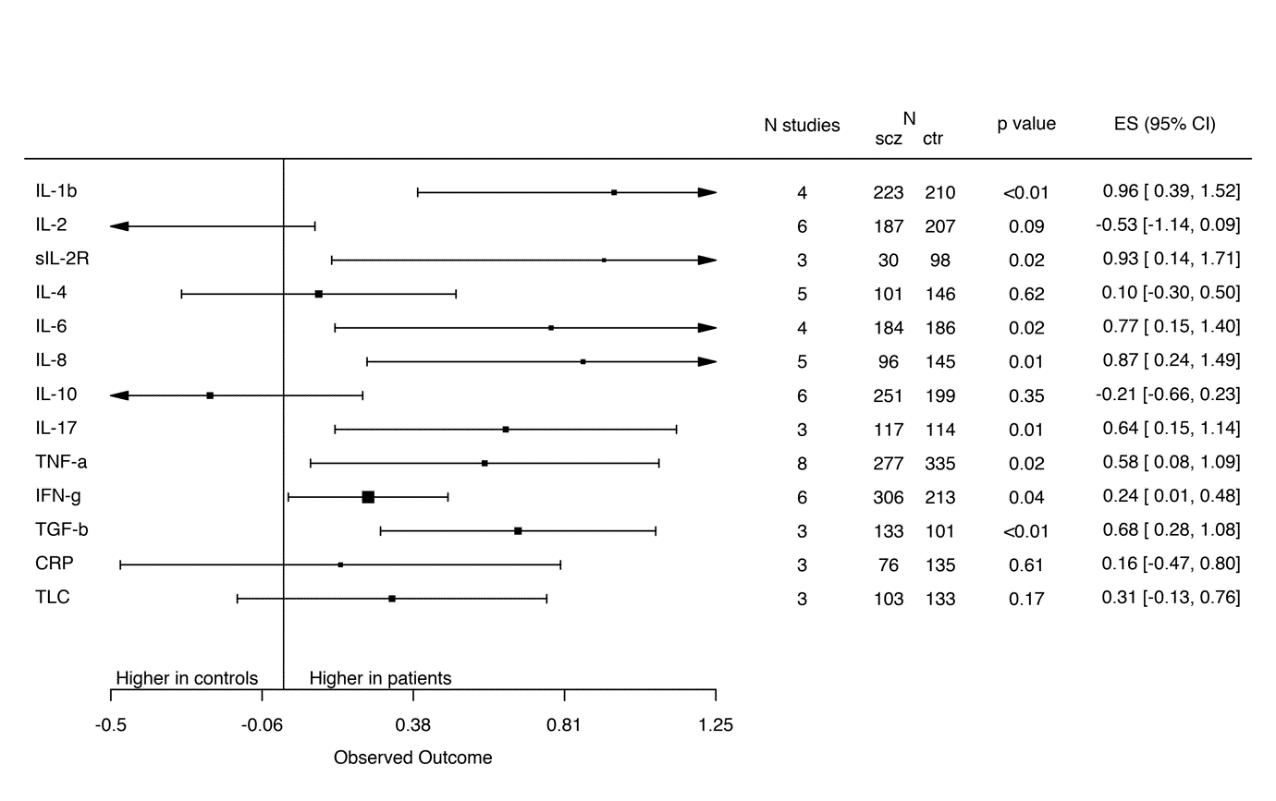
**

**eFigure 4**

**Funnel plot of standard error and residual value (individual estimate minus pooled estimate) for Hedges *g* across all immune parameters.**


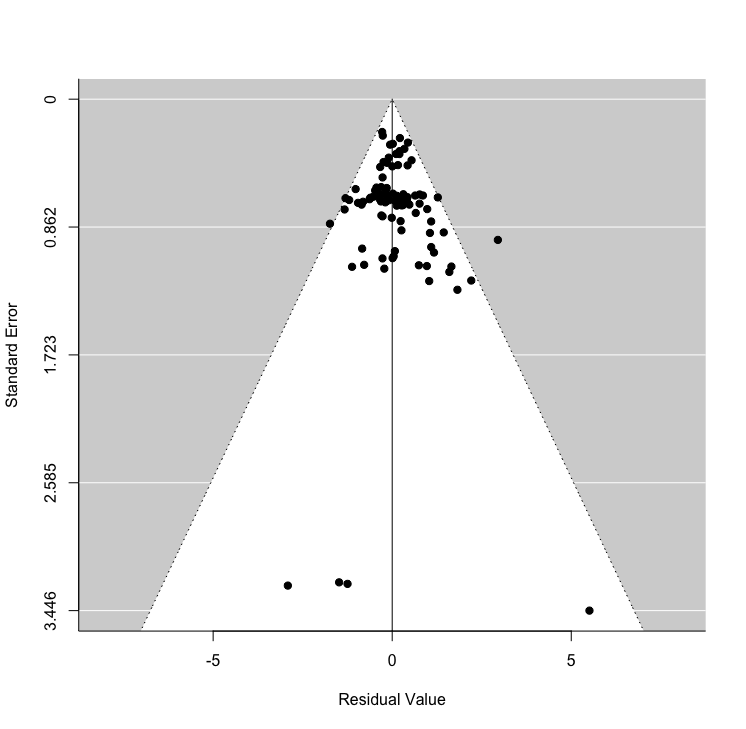


**eFigure 5 (next page)**

**Kernel Density Plots of raw data for immune parameters in antipsychotic naïve first episode psychosis compared with healthy controls. Each line represents a different study, with studies examining the same immune parameter drawn on the same Density Plot, data having been normalised (scaled to mean).**


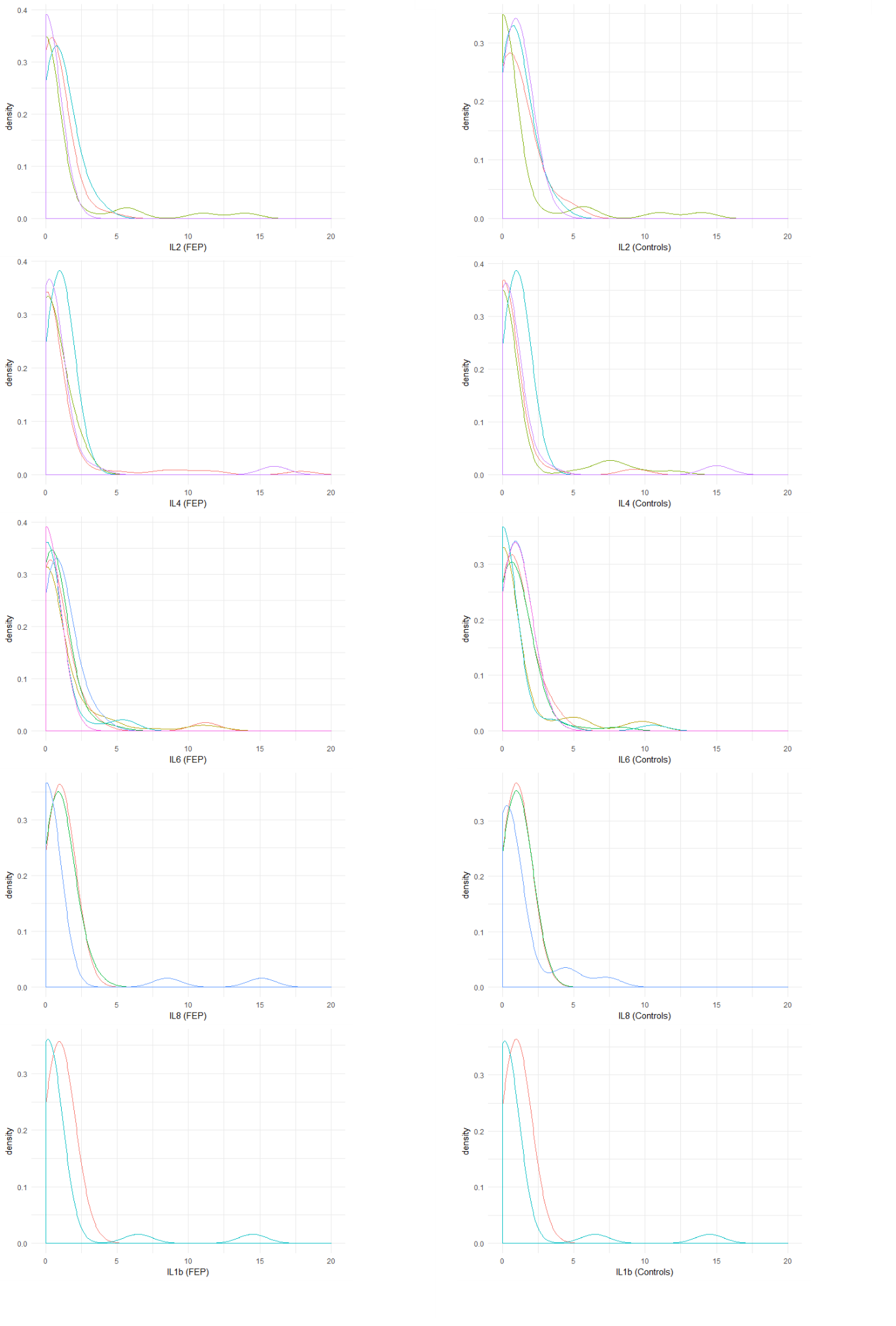


**
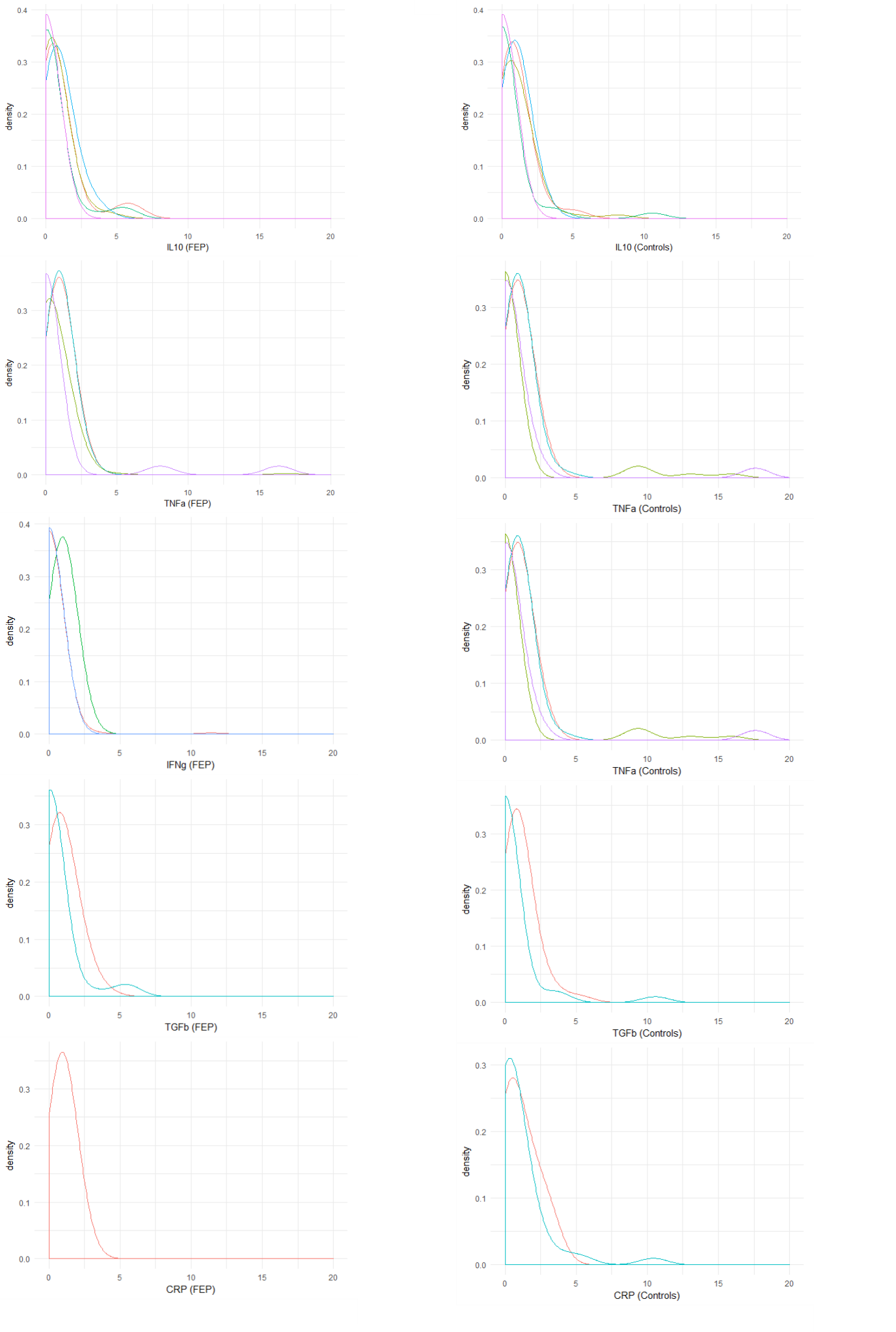
**

**eTable 1**

**Raw data set used in meta-analysis**

| **Parameter** | **Author** | **Year** | **FEP**  **N** | **FEP Mean** | **FEP**  **SD** | **HC N** | **HC**  **Mean** | **HC**  **SD** |
| --- | --- | --- | --- | --- | --- | --- | --- | --- |
| tnfa | Ajami | 2014 | 8 | 26.12 | 15.09 | 26 | 29.007 | 5.21 |
| il10 | Ajami | 2014 | 5 | 5.14 | 6.6 | 26 | 10.716 | 7.207 |
| il2 | Ajami | 2014 | 8 | 0.12 | 0.11 | 26 | 0.071 | 0.003 |
| il6 | Akiyama | 1999 | 14 | 2165 | 2029 | 27 | 999 | 495 |
| sil2r | Akiyama | 1999 | 14 | 739.3 | 217.2 | 27 | 520.9 | 162.4 |
| tgfb | Borovcanin | 2012 | 84 | 50.88 | 41.07 | 35 | 23.53 | 20.51 |
| crp | De Berardis | 2013 | 30 | 2.8 | 0.9 | 30 | 1.5 | 0.6 |
| crp | Devanarayanan | 2016 | 30 | 305.63 | 140 | 40 | 230.81 | 181.27 |
| il1b | Di Nicola | 2013 | 30 | 17.57 | 29.45 | 24 | 2.1 | 1.959592 |
| il2 | Di Nicola | 2013 | 30 | 50.21 | 97.11 | 24 | 4.5 | 6.368673 |
| il4 | Di Nicola | 2013 | 30 | 3.73 | 1.31 | 24 | 4.6 | 3.919184 |
| il6 | Di Nicola | 2013 | 30 | 3.76 | 5.83 | 24 | 1.5 | 2.44949 |
| il8 | Di Nicola | 2013 | 30 | 804.98 | 1237.19 | 24 | 71.9 | 106.7978 |
| il10 | Di Nicola | 2013 | 30 | 2.12 | 1.55 | 24 | 1.9 | 2.939388 |
| tnfa | Di Nicola | 2013 | 5 | 54.88 | 109.18 | 24 | 15.9 | 24.4949 |
| ifng | Di Nicola | 2013 | 5 | 1.5 | 0.83 | 24 | 3.7 | 8.818163 |
| il6 | Ding | 2014 | 69 | 14.75 | 4.52 | 60 | 11.76 | 5.05 |
| il17 | Ding | 2014 | 69 | 17.69 | 6.73 | 60 | 15.61 | 5.46 |
| ifng | Ding | 2014 | 69 | 509.73 | 144.44 | 60 | 438.54 | 113.7 |
| ifng | El Kissi | 2015 | 8 | 31.14 | 36.5 | 21 | 23.03 | 16.85 |
| tgfb | El Kissi | 2015 | 10 | 7039.2 | 3638.71 | 27 | 7082.28 | 7466.91 |
| il17 | El Kissi | 2015 | 9 | 246 | 302.51 | 15 | 36.07 | 43.16 |
| crp | Fawzi | 2011 | 108 | 3.2 | 1.6 | 200 | 1.4 | 0.7 |
| crp | Fernandez-Egea | 2009 | 50 | 0.21 | 0.28 | 50 | 0.2 | 0.18 |
| il6 | Fernandez-Egea | 2009 | 50 | 3.63 | 6.91 | 50 | 1.02 | 2.1 |
| il2 | Ganguli | 1995 | 33 | 935.8 | 646.9 | 33 | 1566.5 | 832.1 |
| il6 | Ganguli | 1994 | 24 | 0.6265354 | 0.916998 | 110 | 0.6479447 | 1.009718 |
| wcc | Garcia-Rizo | 2017 | 75 | 2.3 | 0.8 | 80 | 2.23 | 0.6 |
| il2 | Gattaz | 1992 | 10 | 0.49 | 0.37 | 11 | 0.69 | 0.61 |
| ifng | Gattaz | 1992 | 10 | 20.8 | 32.4 | 15 | 13 | 16.9 |
| il1b | Haring | 2015 | 38 | 1.35 | 0.7 | 37 | 1.88 | 0.89 |
| il2 | Haring | 2015 | 38 | 3 | 0.65 | 37 | 2.85 | 0.73 |
| il4 | Haring | 2015 | 38 | 1.73 | 0.55 | 37 | 1.4 | 0.37 |
| il6 | Haring | 2015 | 38 | 1.35 | 1.14 | 37 | 0.66 | 0.43 |
| il8 | Haring | 2015 | 38 | 5.89 | 3.74 | 37 | 5.51 | 2.8 |
| il10 | Haring | 2015 | 38 | 0.62 | 0.35 | 37 | 0.6 | 0.36 |
| ifng | Haring | 2015 | 38 | 0.35 | 0.13 | 37 | 0.3 | 0.09 |
| tnfa | Haring | 2015 | 38 | 2.04 | 0.93 | 37 | 2.16 | 1.41 |
| crp | Hepgul | 2012 | 4 | 0.11 | 0.12 | 45 | 0.24 | 0.44 |
| il6 | Kalmady | 2014 | 25 | 2.2 | 1.8 | 33 | 1.4 | 0.8 |
| il6 | Kubistova | 2012 | 25 | 14.108 | 31.97 | 25 | 1.438 | 1.366 |
| il8 | Kubistova | 2012 | 25 | 4.745 | 2.168 | 25 | 4.438 | 1.862 |
| il10 | Kubistova | 2012 | 25 | 19.194 | 29.118 | 25 | 8.845 | 8.986 |
| tnfa | Kubistova | 2012 | 25 | 6.023 | 3.064 | 25 | 3.768 | 2.287 |
| wcc | Masserini | 1990 | 7 | 2251 | 717 | 37 | 1958 | 62 |
| il1b | Mondelli | 2015 | 3 | 88.48 | 145.11 | 36 | 2.5203 | 2.34393 |
| il2 | Mondelli | 2015 | 3 | 3.53 | 2.19 | 36 | 3.2539 | 2.16089 |
| il4 | Mondelli | 2015 | 3 | 3.4 | 1.91 | 36 | 3.7137 | 1.08789 |
| il6 | Mondelli | 2015 | 3 | 463.06 | 799.96 | 36 | 0.9433 | 0.55842 |
| il8 | Mondelli | 2015 | 3 | 1172.15 | 1465.22 | 36 | 44.3464 | 55.05175 |
| il10 | Mondelli | 2015 | 3 | 15.95 | 24.51 | 36 | 1.3294 | 0.86636 |
| tnfa | Mondelli | 2015 | 3 | 455.8 | 277.25 | 36 | 6.4497 | 2.56009 |
| ifng | Mondelli | 2015 | 3 | 1.33 | 0.46 | 36 | 1.6161 | 1.359 |
| sil2r | Rapoport | 1994 | 12 | 1705.7 | 1124.2 | 14 | 739.8 | 325.5 |
| il2 | Simsek | 2016 | 30 | 20 | 13 | 26 | 13.7 | 11.3 |
| il4 | Simsek | 2016 | 30 | 30.5 | 5.3 | 26 | 30.1 | 5.7 |
| il6 | Simsek | 2016 | 30 | 4.3 | 9.9 | 26 | 2.6 | 7.2 |
| il10 | Simsek | 2016 | 30 | 5.1 | 3.6 | 26 | 7.4 | 5 |
| il17 | Simsek | 2016 | 30 | 7.6 | 16.2 | 26 | 1.9 | 6.8 |
| tnfa | Simsek | 2016 | 30 | 9.2 | 22.2 | 26 | 4.2 | 4.9 |
| ifng | Simsek | 2016 | 30 | 2.9 | 15.6 | 26 | 1.03 | 7.1 |
| wcc | Sperner-Unterweger | 1999 | 21 | 2736 | 1216 | 16 | 2121 | 649 |
| il1b | Song | 2013 | 62 | 53.28 | 12.62 | 60 | 23.49 | 15.27 |
| il6 | Song | 2013 | 62 | 33.98 | 14.13 | 60 | 15.53 | 7.16 |
| tnfa | Song | 2013 | 62 | 50.08 | 12.86 | 60 | 32.12 | 14.74 |
| il1b | Song | 2009 | 83 | 25.93 | 13.3 | 65 | 14.19 | 7.86 |
| tnfa | Song | 2009 | 83 | 31.01 | 13.82 | 65 | 21.33 | 8.9 |
| il2 | Theodoropoulou | 2001 | 53 | 220 | 25 | 62 | 271 | 50 |
| il1b | Theodoropoulou | 2001 | 53 | 21.4 | 8 | 62 | 12 | 6 |
| tnfa | Theodoropoulou | 2001 | 53 | 24 | 8 | 62 | 16 | 6 |
| il10 | Xiu | 2014 | 128 | 39.2 | 25.4 | 62 | 51.2 | 36.6 |
| sil2r | Sirota | 2005 | 6 | 1760 | 325.3 | 22 | 1114.6 | 316.6 |
| il6 | Noto | 2015-16 | 156 | 3.23 | 15.32 | 58 | 0.77 | 1.04 |
| sil2r | Ganguli | 1989 | 4 | 294.7 | 58.2 | 57 | 343.8 | 138.9 |
| il4 | Borovcanin | 2012 | 63 | 27.42 | 84.84 | 37 | 12.09 | 47.34 |
| il6 | Borovcanin | 2012 | 87 | 29.42 | 69.51 | 37 | 8.14 | 20.56 |
| il17 | Borovcanin | 2012 | 85 | 9.76 | 47.67 | 37 | 26.69 | 71.69 |
| tgfb | Petrikis | 2015 | 39 | 112.29 | 45.35 | 39 | 92.71 | 54.51 |
| il6 | Petrikis | 2015 | 39 | 0.92 | 3.35 | 39 | 0.12 | 0.43 |
| il17 | Petrikis | 2015 | 39 | 0.64 | 3.13 | 39 | 0.57 | 2.66 |
| il4 | Noto | 2015-16 | 156 | 1.05 | 5.75 | 57 | 0.23 | 0.63 |
| il10 | Noto | 2015-16 | 156 | 0.9 | 2.53 | 57 | 0.2 | 0.45 |
| il17 | Noto | 2015-16 | 156 | 2.93 | 5.43 | 57 | 4.25 | 11.87 |
| tnfa | Noto | 2015-16 | 156 | 1.18 | 4.48 | 57 | 0.15 | 0.51 |
| ifng | Noto | 2015-16 | 156 | 0.06 | 0.37 | 57 | 0.02 | 0.16 |
| il6 | Karanikas | 2017 | 25 | 484.5 | 2290.2 | 23 | 4.08 | 19.13 |
| il17 | Karanikas | 2017 | 25 | 768 | 3127 | 23 | 61 | 204 |
| il1b | Karanikas | 2017 | 25 | 513.6 | 1586.7 | 23 | 87.3 | 101.9 |
| il2 | Karanikas | 2017 | 25 | 1178.4 | 4275.9 | 23 | 115.5 | 72 |
| il4 | Karanikas | 2017 | 25 | 296.4 | 942.5 | 23 | 79.3 | 37.8 |
| il8 | Karanikas | 2017 | 25 | 6281.1 | 21322 | 23 | 1480 | 2727 |
| il10 | Karanikas | 2017 | 25 | 8597 | 41520 | 23 | 70 | 197 |
| ifng | Karanikas | 2017 | 25 | 17670 | 84658 | 23 | 127 | 388 |
| tnfa | Karanikas | 2017 | 25 | 1230 | 4398 | 23 | 31 | 114 |

**eTable 2**

**Immune parameter skew ratios for patients compared with controls: P values associated with Fisher’s Exact test**

|  | | **Raw scaled** | | **Log transformed** | |
| --- | --- | --- | --- | --- | --- |
| **Parameter** | **Total number of data points (patients + controls)** | **Number of severely skewed data points** | **Fisher’s Exact Test: Proportion of skewed data in patients VS controls (p value)** | **Number of severely skewed data points** | **Fisher’s Exact Test: Proportion of skewed data in patients VS controls (p value)** |
| CRP | 10 | 3 | 0.40 | 1 | 0.44 |
| IFNγ | 18 | 10 | 0.99 | 6 | 0.99 |
| IL10 | 18 | 9 | 0.99 | 5 | 0.99 |
| IL17 | 14 | 12 | 0.99 | 4 | 0.99 |
| IL1β | 14 | 4 | 0.56 | 4 | 0.56 |
| IL2 | 18 | 3 | 0.99 | 3 | 0.21 |
| IL4 | 14 | 5 | 0.99 | 3 | 0.99 |
| IL6 | 30 | 18 | 0.71 | 22 | 0.26 |
| IL8 | 10 | 6 | 0.99 | 0 | 0.99 |
| sIL2R | 8 | 0 | 0.99 | 1 | 0.99 |
| TGFβ | 6 | 1 | 0.20 | 0 | 0.99 |
| TNFα | 22 | 8 | 0.99 | 4 | 0.59 |
| TLC | 6 | 0 | 0.99 | 0 | 0.99 |

**eTable 3**

**Quality assessment of studies included in meta-analysis.**

The Newcastle Ottawa Scale considers study quality based on three domains (see eAppendix 3 for further details): 1) Quality of subject ‘selection’ (cases and controls, maximum 4 points); 2) Quality of ‘comparability’ of cases and controls (defined a priori as matching for age and BMI, maximum 2 points); 3) Quality of ‘exposure’ (defined as use of structured interview to define patients as experiencing a first episode of psychosis or use of DSM/ICD diagnoses of schizophrenia, schizoaffective disorder, schizophreniform disorder, schizophrenia spectrum or psychotic disorder not otherwise specified, and use of structured interview to define absence of mental illness in health controls, maximum 2 points). Item ‘non-response rate’ for Quality of exposure in the scale was not applicable. A maximum of 8 points was therefore considered.

| **Author/Year** | **Selection** | | | | **Comparability** | | **Exposure** | | **Score**  **/8** | **Quality Rating** |
| --- | --- | --- | --- | --- | --- | --- | --- | --- | --- | --- |
|  | **1** | **2** | **3** | **4** | **Age** | **BMI** | **1** | **2** |  |  |
| Ajami et al., 2014^5^ | 1 | 0 | 1 | 1 | 0 | 0 | 1 | 0 | 4 | Fair |
| Akiyama et al., 1999^6^ | 1 | 0 | 0 | 1 | 1 | 0 | 1 | 0 | 4 | Fair |
| Borovcanin et al., 2012^7^ | 1 | 1 | 0 | 1 | 1 | 0 | 1 | 0 | 4 | Good |
| De Berardis et al., 2012^8^ | 1 | 1 | 0 | 0 | 1 | 0 | 1 | 0 | 4 | Fair |
| Devanarayanan et al., 2016^9^ | 1 | 1 | 0 | 1 | 1 | 0 | 1 | 0 | 5 | Good |
| Di Nicola et al., 2013^10^ | 1 | 1 | 0 | 1 | 1 | 1 | 1 | 0 | 6 | Good |
| Ding et al., 2014^11^ | 1 | 1 | 1 | 1 | 1 | 1 | 1 | 1 | 8 | Good |
| El Kissi et al., 2015^12^ | 1 | 1 | 1 | 1 | 1 | 0 | 1 | 0 | 6 | Good |
| Fawzi et al., 2011^13^ | 1 | 1 | 1 | 1 | 1 | 1 | 1 | 1 | 8 | Good |
| Fernandez-Egea et al., 2009^14^ | 1 | 1 | 1 | 1 | 1 | 1 | 1 | 1 | 8 | Good |
| Ganguli et al., 1989^15^ | 0 | 0 | 0 | 0 | 0 | 0 | 0 | 0 | 0 | Poor |
| Ganguli et al., 1994^16^ | 1 | 1 | 1 | 1 | 1 | 0 | 1 | 0 | 6 | Good |
| Ganguli et al., 1995^17^ | 1 | 1 | 1 | 1 | 1 | 0 | 1 | 1 | 7 | Good |
| Garcia-Rizo et al., 2017^18^ | 1 | 1 | 1 | 1 | 1 | 1 | 1 | 1 | 8 | Good |
| Gattaz et al., 1992^19^ | 1 | 0 | 0 | 1 | 1 | 0 | 1 | 0 | 4 | Fair |
| Haring et al., 2015^20^  Balotsev et al., 2017^21^ | 1 | 1 | 1 | 1 | 1 | 1 | 1 | 1 | 8 | Good |
| Hepgul et al., 2012^22^ | 1 | 1 | 1 | 1 | 0 | 0 | 1 | 1 | 6 | Poor |
| Kalmady et al., 2014^23^ | 1 | 1 | 1 | 0 | 1 | 0 | 1 | 1 | 6 | Good |
| Karanikas et al., 2017^24^ | 1 | 1 | 0 | 1 | 1 | 1 | 1 | 1 | 7 | Good |
| Kubistova et al., 2012^25^ | 1 | 1 | 0 | 1 | 1 | 0 | 1 | 1 | 7 | Good |
| Masserini et al., 1990^26^ | 1 | 0 | 1 | 1 | 0 | 0 | 1 | 0 | 4 | Poor |
| Mondelli et al., 2015^27^ | 1 | 1 | 0 | 1 | 1 | 0 | 1 | 1 | 6 | Good |
| Noto et al., 2015^28^  Noto et al., 2016^29^  Brinholi et al., 2015^30^ | 1 | 1 | 0 | 1 | 1 | 0 | 1 | 0 | 5 | Good |
| Petrikis et al., 2015^31^ | 1 | 1 | 0 | 1 | 1 | 1 | 1 | 1 | 7 | Good |
| Rapoport et al., 1994^32^ | 1 | 1 | 1 | 1 | 1 | 0 | 1 | 0 | 6 | Good |
| Simsek et al., 2016^33^ | 1 | 1 | 0 | 1 | 1 | 1 | 1 | 0 | 6 | Good |
| Sperner-Unterweger et al., 1999^34^ | 1 | 1 | 0 | 1 | 0 | 0 | 1 | 0 | 4 | Poor |
| Sirota et al., 2005^35^ | 1 | 1 | 0 | 1 | 1 | 0 | 1 | 0 | 5 | Good |
| Song et al., 2009^36^ | 1 | 1 | 0 | 1 | 1 | 1 | 1 | 0 | 6 | Good |
| Song et al., 2013^37^  Song et al., 2014^38^ | 1 | 1 | 1 | 1 | 1 | 1 | 1 | 0 | 7 | Good |
| Theodoropoulou et al., 2001^39^ | 1 | 1 | 1 | 1 | 1 | 0 | 1 | 0 | 6 | Good |
| Xiu et al., 2014^40^ | 1 | 1 | 1 | 1 | 1 | 1 | 1 | 0 | 7 | Good |

Thresholds used for converting the NOS rating to Agency for Healthcare Research and Quality - AHRQ - standards (good, fair, and poor):

Good quality: 3 or 4 stars in Selection domain AND 1 or 2 stars in Comparability domain AND 1 or 2 stars in Outcome domain

Fair quality: 2 stars in Selection domain AND 1 or 2 stars in Comparability domain AND 1 or 2 stars in Outcome domain

Poor quality: 0 or 1 star in Selection domain OR 0 stars in Comparability domain OR 0 or 1 stars in Outcome domain

**eTable 4**

**I^2^ values for each immune parameter (standardised difference in means analysis)**

| **Parameter** | **Study N** | **I^2^ value** |
| --- | --- | --- |
| IL1β | 7 | 90.69 |
| IL2 | 9 | 81.88 |
| sIL2r | 4 | 96.98 |
| IL4 | 7 | 56.50 |
| IL6 | 15 | 83.64 |
| IL8 | 5 | 81.86 |
| IL10 | 9 | 81.92 |
| IL17 | 7 | 84.93 |
| TNFα | 11 | 82.30 |
| IFNγ | 9 | 37.42 |
| TGFβ | 3 | 35.51 |
| CRP | 5 | 92.17 |
| TLC | 3 | 63.95 |

**eTable 5**

Hartigan’s Dip Test of unimodality for a subgroup of raw data sets included in the meta-analysis. A p value < 0.05 indicates the data distribution is likely to be multi-modal.

| **Study** | **Parameter** | **FEP/Control (n)** | **Hartigan’s Dip Test** | **Modality** |
| --- | --- | --- | --- | --- |
| Haring et al., 2015^1^  Balotsev et al., 2017^2^ | IL1β | FEP (38) | D = 0.06;  p = 0.24 | Unimodal |
| Karanikas et al., 2017^3^ | IL1β | FEP (25) | D = 0.04;  p = 0.88 | Unimodal |
| Haring et al., 2015^1^  Balotsev et al., 2017^2^ | IL1β | Control (37) | D = 0.03;  p = 0.99 | Unimodal |
| Karanikas et al., 2017^3^ | IL1β | Control (23) | D = 0.06;  p = 0.79 | Unimodal |
|  | | | | |
| Noto et al., 2015^4^  Noto et al., 2016^5^  Brinholi et al., 2015^6^ | IL2 | FEP (156) | D = 0.01;  p = 1.00 | Unimodal |
| Petrikis et al., 2015^7^ | IL2 | FEP (39) | D = 0.03;  p = 1.00 | Unimodal |
| Haring et al., 2015^1^  Balotsev et al., 2017^2^ | IL2 | FEP (38) | D = 0.07;  p = 0.25 | Unimodal |
| Karanikas et al., 2017^3^ | IL2 | FEP (25) | D = 0.04;  p = 1.00 | Unimodal |
| Noto et al., 2015^4^  Noto et al., 2016^5^  Brinholi et al., 2015^6^ | IL2 | Control (58) | D = 0.01;  p = 1.00 | Unimodal |
| Petrikis et al., 2015^7^ | IL2 | Control (39) | D = 0.01;  p = 1.00 | Unimodal |
| Haring et al., 2015^1^  Balotsev et al., 2017^2^ | IL2 | Control (37) | D = 0.07;  p = 0.14 | Unimodal |
| Karanikas et al., 2017^3^ | IL2 | Control (23) | D = 0.06;  p = 0.66 | Unimodal |
|  | | | | |
| Borovcanin et al., 2012^8^ | IL4 | FEP (84) | D = 0.02;  P = 1.00 | Unimodal |
| Noto et al., 2015^4^  Noto et al., 2016^5^  Brinholi et al., 2015^6^ | IL4 | FEP (156) | D = 0.04;  P = 0.14 | Unimodal |
| Haring et al., 2015^1^  Balotsev et al., 2017^2^ | IL4 | FEP (38) | D = 0.07  P = 0.20 | Unimodal |
| Karanikas et al., 2017^3^ | IL4 | FEP (25) | D = 0.04;  p = 0.99 | Unimodal |
| Borovcanin et al., 2012^8^ | IL4 | Control (35) | D = 0.02;  p = 1.00 | Unimodal |
| Noto et al., 2015^4^  Noto et al., 2016^5^  Brinholi et al., 2015^6^ | IL4 | Control (58) | D = 0.03;  p = 0.98 | Unimodal |
| Haring et al., 2015^1^  Balotsev et al., 2017^2^ | IL4 | Control (37) | D = 0.05;  p = 0.56 | Unimodal |
| Karanikas et al., 2017^3^ | IL4 | Control (23) | D = 0.05;  p = 0.88 | Unimodal |
|  | | | | |
| Kubistova et al., 2012^9^ | IL6 | FEP (25) | D = 0.04;  p = 0.99 | Unimodal |
| Borovcanin et al., 2012^8^ | IL6 | FEP (84) | D = 0.03;  p = 0.90 | Unimodal |
| Noto et al., 2015^4^  Noto et al., 2016^5^  Brinholi et al., 2015^6^ | IL6 | FEP (156) | D = 0.03;  p < 0.05 | Non-unimodal |
| Petrikis et al., 2015^7^ | IL6 | FEP (39) | D = 0.04;  p = 0.91 | Unimodal |
| Haring et al., 2015^1^  Balotsev et al., 2017^2^ | IL6 | FEP (38) | D = 0.04;  p = 0.81 | Unimodal |
| Karanikas et al., 2017^3^ | IL6 | FEP (25) | D = 0.02;  p = 1.00 | Unimodal |
| Kubistova et al., 2012^9^ | IL6 | Control (25) | D = 0.05;  p = 0.96 | Unimodal |
| Borovcanin et al., 2012^8^ | IL6 | Control (35) | D = 0.03;  p = 1.00 | Unimodal |
| Noto et al., 2015^4^  Noto et al., 2016^5^  Brinholi et al., 2015^6^ | IL6 | Control (58) | D = 0.04;  p = 0.53 | Unimodal |
| Petrikis et al., 2015^7^ | IL6 | Control (39) | D = 0.02;  p = 1.00 | Unimodal |
| Haring et al., 2015^1^  Balotsev et al., 2017^2^ | IL6 | Control (37) | D = 0.06;  p = 0.47 | Unimodal |
| Karanikas et al., 2017^3^ | IL6 | Control (23) | D = 0.02;  p = 1.00 | Unimodal |
|  | | | | |
| Kubistova et al., 2012^9^ | IL8 | FEP (25) | D = 0.05;  p = 0.87 | Unimodal |
| Haring et al., 2015^1^  Balotsev et al., 2017^2^ | IL8 | FEP (38) | D = 0.04;  p = 0.98 | Unimodal |
| Karanikas et al., 2017^3^ | IL8 | FEP (25) | D = 0.05;  p = 0.88 | Unimodal |
| Kubistova et al., 2012^9^ | IL8 | Control (25) | D = 0.05;  p = 0.84 | Unimodal |
| Haring et al., 2015^1^  Balotsev et al., 2017^2^ | IL8 | Control (37) | D = 0.05  p = 0.52 | Unimodal |
| Karanikas et al., 2017^3^ | IL8 | Control (23) | D = 0.05;  p = 0.96 | Unimodal |
|  | | | | |
| Kubistova et al., 2012^9^ | IL10 | FEP (25) | D = 0.06;  p = 0.63 | Unimodal |
| Petrikis et al., 2015^7^ | IL10 | FEP (39) | D = 0.01;  p = 1.00 | Unimodal |
| Haring et al., 2015^1^  Balotsev et al., 2017^2^ | IL10 | FEP (38) | D = 0.07;  p = 0.17 | Unimodal |
| Karanikas et al., 2017^3^ | IL10 | FEP (25) | D = 0.06;  p = 0.66 | Unimodal |
| Kubistova et al., 2012^9^ | IL10 | Control (25) | D = 0.04;  p = 0.99 | Unimodal |
| Petrikis et al., 2015^7^ | IL10 | Control (39) | D = 0.01;  p = 1.00 | Unimodal |
| Haring et al., 2015^1^  Balotsev et al., 2017^2^ | IL10 | Control (37) | D = 0.06;  p = 0.45 | Unimodal |
| Karanikas et al., 2017^3^ | IL10 | Control (23) | D = 0.07;  p = 0.61 | Unimodal |
|  | | | | |
| Borovcanin et al., 2012^8^ | TGFβ | FEP (84) | D = 0.03  p = 0.97 | Unimodal |
| Petrikis et al., 2015^7^ | TGFβ | FEP (39) | D = 0.04;  p = 0.88 | Unimodal |
| Borovcanin et al., 2012^8^ | TGFβ | Control (35) | D = 0.03;  p = 1.00 | Unimodal |
| Petrikis et al., 2015^7^ | TGFβ | Control (39) | D = 0.02;  p = 1.00 | Unimodal |
|  | | | | |
| Kubistova et al., 2012^9^ | TNFα | FEP (25) | D = 0.05;  p = 0.83 | Unimodal |
| Haring et al., 2015^1^  Balotsev et al., 2017^2^ | TNFα | FEP (38) | D = 0.04;  p = 0.94 | Unimodal |
| Karanikas et al., 2017^3^ | TNFα | FEP (25) | D = 0.06;  p = 0.64 | Unimodal |
| Kubistova et al., 2012^9^ | TNFα | Control (25) | D = 0.05;  p = 0.90 | Unimodal |
| Haring et al., 2015^1^  Balotsev et al., 2017^2^ | TNFα | Control (37) | D = 0.03;  p = 0.99 | Unimodal |
| Karanikas et al., 2017^3^ | TNFα | Control (23) | D = 0.03;  p = 1.00 | Unimodal |
|  | | | | |
| Noto et al., 2015^4^  Noto et al., 2016^5^  Brinholi et al., 2015^6^ | IFNγ | FEP (156) | D = 0.01;  p = 1.00 | Unimodal |
| Haring et al., 2015^1^  Balotsev et al., 2017^2^ | IFNγ | FEP (38) | D = 0.10;  p < 0.05 | Non-unimodal |
| Karanikas et al., 2017^3^ | IFNγ | FEP (25) | D = 0.07;  p = 0.45 | Unimodal |
| Noto et al., 2015^4^  Noto et al., 2016^5^  Brinholi et al., 2015^6^ | IFNγ | Control (58) | D = 0.01;  p = 1.00 | Unimodal |
| Haring et al., 2015^1^  Balotsev et al., 2017^2^ | IFNγ | Control (37) | D = 0.08;  p = 0.07 | Unimodal |
| Karanikas et al., 2017^3^ | IFNγ | Control (23) | D = 0.06;  p = 0.86 | Unimodal |
|  | | | | |
| Devanarayanan et al., 2016^10^ | CRP | FEP (22) | D = 0.07;  p = 0.53 | Unimodal |
| Hepgul et al., 2012^11^ | CRP | Control (45) | D = 0.07;  P = 0.12 | Unimodal |
| Devanarayanan et al., 2016^10^ | CRP | Control (40) | D = 0.04;  P = 0.97 | Unimodal |

**eTable 6**

**Immune parameter measurement techniques**

| **Study** | **Assay** |
| --- | --- |
| Ajami et al., 2014^5^ | Enzyme-linked immunosorbent assay |
| Akiyama et al., 1999^6^ | Sandwich immunoassay |
| Borovcanin et al., 2012^7^ | Enzyme-linked immunosorbent assay |
| De Berardis et al., 2012^8^ | Nephelometric assay |
| Devanarayanan et al., 2016^9^ | Enzyme-linked immunosorbent assay |
| Di Nicola et al., 2013^10^ | Sandwich immunoassay |
| Ding et al., 2014^11^ | Enzyme-linked immunosorbent assay |
| El Kissi et al., 2015^12^ | Enzyme-linked immunosorbent assay |
| Fawzi et al., 2011^13^ | Immunoturbidimetric assay |
| Fernandez-Egea et al., 2009^14^ | Not described |
| Ganguli et al., 1989^15^ | Enzyme-linked immunosorbent assay |
| Ganguli et al., 1994^16^ | Sandwich Immunoassay |
| Ganguli et al., 1995^17^ | Enzyme-linked immunosorbent assay |
| Garcia-Rizo et al., 2017^18^ | Advia120 cell counter |
| Gattaz et al., 1992^19^ | Enzyme-linked immunosorbent assay |
| Haring et al., 2015^20^  Balotsev et al., 2017^21^ | High-sensitive biochip array technology |
| Hepgul et al., 2012^22^ | Anti-CRP antibody sensitized to latex particles |
| Kalmady et al., 2014^23^ | Sandwich immunoassay |
| Karanikas et al., 2017^24^ | Fluorescent Bead Immunoassay and the Flow Cytomix |
| Kubistova et al., 2012^25^ | Luminex assay |
| Masserini et al., 1990^26^ | microscopical examination |
| Mondelli et al., 2015^27^ | Sandwich immunoassay |
| Noto et al., 2015^28^  Noto et al., 2016^29^  Brinholi et al., 2015^30^ | Flow cytometry |
| Petrikis et al., 2015^31^ | Enzyme-linked immunosorbent assay |
| Rapoport et al., 1994^32^ | Sandwich immunoassay |
| Simsek et al., 2016^33^ | cytokine bead array |
| Sperner-Unterweger et al., 1999^34^ | Flow cytometry |
| Sirota et al., 2005^35^ | Sandwich immunoassay |
| Song et al., 2009^36^ | Sandwich immunoassay |
| Song et al., 2013^37^  Song et al., 2014^38^ | Enzyme-linked immunosorbent assay |
| Theodoropoulou et al., 2001^39^ | Enzyme-linked immunosorbent assay |
| Xiu et al., 2014^40^ | Enzyme-linked immunosorbent assay |

**eTable 7**

**Comparison of current meta-analytic findings with previous meta-analytic evidence of peripheral immune alterations in first episode psychosis.**

| **Para**  **meter** | **Miller, 2011**^41^ | | | | **Upthegrove, 2014**^42^ | | | | **Goldsmith, 2016**^43^ | | | | **CURRENT META-ANALYSIS** | | | |
| --- | --- | --- | --- | --- | --- | --- | --- | --- | --- | --- | --- | --- | --- | --- | --- | --- |
|  | Study N | Patient N | Control N | ES | Study N | Patient N | Control N | ES | Study N | Patient N | Control N | ES | Study N | Patient N | Control N | ES  Log transformed (MVA) |
|  | MEDICATED + NAIVE | | | | NAÏVE ONLY | | | | MEDICATED + NAIVE | | | | NAÏVE ONLY | | | |
| IFNγ | 2 | 48 | 189 | 0.57  P < 0.01 | 3 | 103 | 73 | 0.63  P = 0.76 | 7 | 452 | 747 | 0.23  P < 0.01 | 8 | 188 | 242 | 0.32  P <  0.01 |
| IL10 | x | x | x | x | x | x | x | x | 4 | 357 | 461 | 0.18  P = 0.01 | 8 | 259 | 259 | 0.24  P =  0.20 |
| IL17 | x | x | x | x | x | x | x | x | 2 | 157 | 96 | 0.00  P = 0.99 | 3 | 108 | 101 | 0.48  P = 0.03 |
| IL1β | 3 | 151 | 152 | 0.60  P < 0.01 | 3 | 141 | 151 | 1.17  P < 0.01 | 6 | 333 | 298 | 1.25  P < 0.01 | 7 | 269 | 307 | 0.49  P =  0.12 |
| IL2 | 4 | 116 | 276 | -0.09  P = 0.44 | 3 | 68 | 101 | -0.20  P = 0.77 | 5 | 140 | 300 | 0.08  P = 0.48 | 9 | 205 | 278 | -0.07  P =  0.77 |
| IL4 | x | x | x | x | 2 | 93 | 58 | 0.20  P = 0.86 | 4 | 193 | 322 | -0.63 P < 0.01 | 5 | 101 | 146 | 0.23  P =  0.10 |
| IL6 | 4 | 117 | 275 | 1.40  P < 0.01 | 5 | 181 | 247 | 2.21  P = 0.01 | 11 | 506 | 577 | 1.16  P < 0.01 | 12 | 501 | 546 | 0.62  P <  0.0001 |
| IL8 | x | x | x | x | x | x | x | x | 2 | 49 | 49 | 1.75 P < 0.01 | 5 | 96 | 145 | 0.04  P =  0.90 |
| sIL2R | 3 | 30 | 97 | 1.03  P < 0.01 | 3 | 32 | 63 | 1.34  P < 0.01 | 3 | 30 | 97 | 1.04 P < 0.01 | 4 | 36 | 120 | 2.66  P =  0.05 |
| TGFβ | 2 | 81 | 262 | 0.48  P = 0.01 | x | x | x | x | 3 | 169 | 298 | 0.58 P < 0.01 | 3 | 133 | 101 | 0.53  P <  0.01 |
| TNFα | 4 | 200 | 323 | 0.81  P < 0.05 | 3 | 141 | 151 | 0.94  P < 0.01 | 9 | 587 | 842 | 0.31 P < 0.01 | 10 | 332 | 384 | 0.56  P <  0.01 |

| **Para**  **meter** | | **Miller, 2013**^44^ | | | | **Fernandes, 2016**^45^ | | | | **CURRENT META-**  **ANALYSIS** | | | |
| --- | --- | --- | --- | --- | --- | --- | --- | --- | --- | --- | --- | --- | --- |
|  | Study N | PT N | CT N | ES | Study N | PT N | CT N | ES | Study N | PT N | CT N | ES Log transf  ormed (MVA) |  |
|  | | NAÏVE ONLY | | | | MEDICATED + NAIVE | | | | NAÏVE ONLY | | | |
| CRP | | x | x | x | x | 6 | 348 | 360 | 0.63 P = 0.04 | 5 | 214 | 365 | 0.66  P =  0.06 |
| TLC | | 2 | 28 | 53 | 0.77 P < 0.01 | x | x | x | x | 3 | 103 | 133 | 0.31  P =  0.17 |

|  | Significant finding based on *P* < 0.05 |
| --- | --- |
|  | No difference between patients and controls based on *P* ≥0.05 |

**SUPPLEMENTARY REFERENCES**

1. Roberts WL. CDC/AHA Workshop on Markers of Inflammation and Cardiovascular Disease - Application to Clinical and Public Health Practice - Laboratory tests available to assess inflammation performance and standardization - A background paper. *Circulation* 2004; **110**(25)**:** E572-E576.

2. Jin W, Dong C. IL-17 cytokines in immunity and inflammation. *Emerg Microbes Infec* 2013; **2**.

3. Lundin K, Tuukkanen AM, Jansson C, Nordstrom T, Lindqvist C. No soluble common cytokine receptor gamma chain (gamma(c)) in activated human lymphocyte cultures-comparison with soluble IL-2Ralpha. *Immunol Lett* 2002; **82**(3)**:** 235-240.

4. Witkowska AM. On the role of sIL-2R measurements in rheumatoid arthritis and cancers. *Mediators Inflamm* 2005; **2005**(3)**:** 121-130.

5. Ajami A, Abedian F, Hamzeh Hosseini S, Akbarian E, Alizadeh-Navaei R, Taghipour M. Serum TNF-alpha, IL-10 and IL-2 in schizophrenic patients before and after treatment with risperidone and clozapine. *Iranian journal of immunology : IJI* 2014; **11**(3)**:** 200-209.

6. Akiyama K. Serum levels of soluble IL-2 receptor alpha, IL-6 and IL-1 receptor antagonist in schizophrenia before and during neuroleptic administration. *Schizophrenia Research* 1999; **37**(1)**:** 97-106.

7. Borovcanin M, Jovanovic I, Radosavljevic G, Djukic Dejanovic S, Bankovic D, Arsenijevic N *et al.* Elevated serum level of type-2 cytokine and low IL-17 in first episode psychosis and schizophrenia in relapse. *J Psychiatr Res* 2012; **46**(11)**:** 1421-1426.

8. De Berardis D, Serroni N, Campanella D, Olivieri L, Ferri F, Marini S *et al.* C-Reactive protein levels and its relationships with suicide risk and alexithymia among newly diagnosed, drug-naive patients with non affective psychosis. *International Clinical Psychopharmacology* 2012; **28:** e35.

9. Devanarayanan S, Nandeesha H, Kattimani S, Sarkar S, Jose J. Elevated copper, hs C-reactive protein and dyslipidemia in drug free schizophrenia: Relation with psychopathology score. *Asian J Psychiatr* 2016; **24:** 99-102.

10. Di Nicola M, Cattaneo A, Hepgul N, Di Forti M, Aitchison KJ, Janiri L *et al.* Serum and gene expression profile of cytokines in first-episode psychosis. *Brain Behavior and Immunity* 2013; **31:** 90-95.

11. Ding M, Song X, Zhao J, Gao J, Li X, Yang G *et al.* Activation of Th17 cells in drug naive, first episode schizophrenia. *Prog Neuropsychopharmacol Biol Psychiatry* 2014; **51:** 78-82.

12. El Kissi Y, Samoud S, Mtiraoui A, Letaief L, Hannachi N, Ayachi M *et al.* Increased Interleukin-17 and decreased BAFF serum levels in drug-free acute schizophrenia. *Psychiatry Res* 2015; **225**(1-2)**:** 58-63.

13. Fawzi MM, Said NS. C-reactive protein serum level in drug-free male Egyptian patients with schizophrenia. *Psychiatry Research* 2011; **190**(1)**:** 91-97.

14. Fernandez-Egea E, Bernardo M, Donner T, Conget I, Parellada E, Justicia A *et al.* Metabolic profile of antipsychotic-naive individuals with non-affective psychosis. *Br J Psychiatry* 2009; **194**(5)**:** 434-438.

15. Ganguli R, Rabin BS. Increased Serum Interleukin-2 Receptor Concentration in Schizophrenic and Brain-Damaged Subjects. *Archives of General Psychiatry* 1989; **46**(3)**:** 292-293.

16. Ganguli R, Yang Z, Shurin G, Chengappa KN, Brar JS, Gubbi AV *et al.* Serum interleukin-6 concentration in schizophrenia: elevation associated with duration of illness. *Psychiatry Res* 1994; **51**(1)**:** 1-10.

17. Ganguli R, Brar JS, Chengappa K, DeLeo M, Yang ZW, Shurin G *et al.* Mitogen-stimulated interleukin-2 production in never-medicated, first-episode schizophrenic patients: The influence of age at onset and negative symptoms. *Archives of General Psychiatry* 1995; **52**(8)**:** 668-672.

18. Garcia-Rizo C, Casanovas M, Fernandez-Egea E, Oliveira C, Meseguer A, Cabrera B *et al.* Blood cell count in antipsychotic-naive patients with non-affective psychosis. *Early Interv Psychiatry* 2017.

19. Gattaz WF, Dalgalarrondo P, Schroder HC. Abnormalities in Serum Concentrations of Interleukin-2, Interferon-Alpha and Interferon-Gamma in Schizophrenia Not Detected. *Schizophrenia Research* 1992; **6**(3)**:** 237-241.

20. Haring L, Koido K, Vasar V, Leping V, Zilmer K, Zilmer M *et al.* Antipsychotic treatment reduces psychotic symptoms and markers of low-grade inflammation in first episode psychosis patients, but increases their body mass index. *Schizophrenia Research* 2015; **169**(1-3)**:** 22-29.

21. Balotsev R, Haring L, Koido K, Leping V, Kriisa K, Zilmer M *et al.* Antipsychotic treatment is associated with inflammatory and metabolic biomarkers alterations among first-episode psychosis patients: A 7-month follow-up study. *Early Interv Psychiatry* 2017.

22. Hepgul N, Pariante CM, Dipasquale S, DiForti M, Taylor H, Marques TR *et al.* Childhood maltreatment is associated with increased body mass index and increased C-reactive protein levels in first-episode psychosis patients. *Psychological Medicine* 2012; **42**(9)**:** 1893-1901.

23. Kalmady SV, Venkatasubramanian G, Shivakumar V, Gautham S, Subramaniam A, Jose DA *et al.* Relationship between Interleukin-6 Gene Polymorphism and Hippocampal Volume in Antipsychotic-Naive Schizophrenia: Evidence for Differential Susceptibility? *Plos One* 2014; **9**(5).

24. Karanikas E, Manganaris S, Ntouros E, Floros G, Antoniadis D, Garyfallos G. Cytokines, cortisol and IGF-1 in first episode psychosis and ultra high risk males. Evidence for TNF-alpha, IFN-gamma, TNF-beta, IL-4 deviation. *Asian Journal of Psychiatry* 2017; **26:** 99-103.

25. Kubistova A, Horacek J, Novak T. Increased interleukin-6 and tumor necrosis factor alpha in first episode schizophrenia patients versus healthy controls. *Psychiatr Danub* 2012; **24 Suppl 1:** S153-156.

26. Masserini C, Vita A, Basile R, Morselli R, Boato P, Peruzzi C *et al.* Lymphocyte Subsets in Schizophrenic Disorders - Relationship with Clinical, Neuromorphological and Treatment Variables. *Schizophrenia Research* 1990; **3**(4)**:** 269-275.

27. Mondelli V, Ciufolini S, Murri MB, Bonaccorso S, Di Forti M, Giordano A *et al.* Cortisol and Inflammatory Biomarkers Predict Poor Treatment Response in First Episode Psychosis. *Schizophrenia Bull* 2015; **41**(5)**:** 1162-1170.

28. Noto C, Ota VK, Santoro ML, Ortiz BB, Rizzo LB, Higuchi CH *et al.* Effects of depression on the cytokine profile in drug naive first-episode psychosis. *Schizophr Res* 2015; **164**(1-3)**:** 53-58.

29. Noto C, Ota VK, Santoro ML, Gouvea ES, Silva PN, Spindola LM *et al.* Depression, Cytokine, and Cytokine by Treatment Interactions Modulate Gene Expression in Antipsychotic Naive First Episode Psychosis. *Mol Neurobiol* 2016; **53**(8)**:** 5701-5709.

30. Brinholi FF, Noto C, Maes M, Bonifacio KL, Brietzke E, Ota VK *et al.* Lowered paraoxonase 1 (PON1) activity is associated with increased cytokine levels in drug naive first episode psychosis. *Schizophr Res* 2015; **166**(1-3)**:** 225-230.

31. Petrikis P, Voulgari PV, Tzallas AT, Archimandriti DT, Skapinakis P, Mavreas V. Cytokine profile in drug-naive, first episode patients with psychosis. *J Psychosom Res* 2015; **79**(4)**:** 324-327.

32. Rapaport MH, Lohr JB. Serum-Soluble Interleukin-2 Receptors in Neuroleptic-Naive Schizophrenic Subjects and in Medicated Schizophrenic Subjects with and without Tardive-Dyskinesia. *Acta Psychiat Scand* 1994; **90**(5)**:** 311-315.

33. Simsek S, Yildirim V, Cim A, Kaya S. Serum IL-4 and IL-10 Levels Correlate with the Symptoms of the Drug-Naive Adolescents with First Episode, Early Onset Schizophrenia. *J Child Adolesc Psychopharmacol* 2016; **26**(8)**:** 721-726.

34. Sperner-Unterweger B, Whitworth A, Kemmler G, Hilbe W, Thaler J, Weiss G *et al.* T-cell subsets in schizophrenia: a comparison between drug-naive first episode patients and chronic schizophrenic patients. *Schizophr Res* 1999; **38**(1)**:** 61-70.

35. Sirota P, Meiman M, Herschko R, Bessler H. Effect of neuroleptic administration on serum levels of soluble IL-2 receptor-alpha and IL-1 receptor antagonist in schizophrenic patients. *Psychiatry Res* 2005; **134**(2)**:** 151-159.

36. Song XQ, Lv LX, Li WQ, Hao YH, Zhao JP. The interaction of nuclear factor-kappa B and cytokines is associated with schizophrenia. *Biol Psychiatry* 2009; **65**(6)**:** 481-488.

37. Song X, Fan X, Song X, Zhang J, Zhang W, Li X *et al.* Elevated levels of adiponectin and other cytokines in drug naive, first episode schizophrenia patients with normal weight. *Schizophr Res* 2013; **150**(1)**:** 269-273.

38. Song X, Fan X, Li X, Zhang W, Gao J, Zhao J *et al.* Changes in pro-inflammatory cytokines and body weight during 6-month risperidone treatment in drug naive, first-episode schizophrenia. *Psychopharmacology (Berl)* 2014; **231**(2)**:** 319-325.

39. Theodoropoulou S, Spanakos G, Baxevanis CN, Economou M, Gritzapis AD, Papamichail MP *et al.* Cytokine serum levels, autologous mixed lymphocyte reaction and surface marker analysis in never medicated and chronically medicated schizophrenic patients. *Schizophrenia Research* 2001; **47**(1)**:** 13-25.

40. Xiu MH, Yang GG, Tan YL, Chen DC, Tan SP, Wang ZR *et al.* Decreased interleukin-10 serum levels in first-episode drug-naive schizophrenia: Relationship to psychopathology. *Schizophrenia Research* 2014; **156**(1)**:** 9-14.

41. Miller BJ, Buckley P, Seabolt W, Mellor A, Kirkpatrick B. Meta-analysis of cytokine alterations in schizophrenia: clinical status and antipsychotic effects. *Biol Psychiatry* 2011; **70**(7)**:** 663-671.

42. Upthegrove R, Manzanares-Teson N, Barnes NM. Cytokine function in medication-naive first episode psychosis: a systematic review and meta-analysis. *Schizophr Res* 2014; **155**(1-3)**:** 101-108 %107 2014/2004/2008 %2018 May %! Cytokine function in medication-naive first episode psychosis: a systematic review and meta-analysis.

43. Goldsmith DR, Rapaport MH, Miller BJ. A meta-analysis of blood cytokine network alterations in psychiatric patients: comparisons between schizophrenia, bipolar disorder and depression. *Mol Psychiatry* 2016.

44. Miller BJ, Gassama B, Sebastian D, Buckley P, Mellor A. Meta-analysis of lymphocytes in schizophrenia: clinical status and antipsychotic effects. *Biol Psychiatry* 2013; **73**(10)**:** 993-999.

45. Fernandes B, Steiner J, Bernstein H, Dodd S, Pasco J, Dean O *et al.* C-reactive protein is increased in schizophrenia but is not altered by antipsychotics: Meta-analysis and implications. *Mol Psychiatr* 2016; **21**(4)**:** 554-564.
